# Fast-forwarding of Hamiltonians and exponentially precise measurements

**DOI:** 10.1038/s41467-017-01637-7

**Published:** 2017-11-17

**Authors:** Yosi Atia, Dorit Aharonov

**Affiliations:** School of Computer Science and Engineering, The Hebrew University of Jerusalem, The Edmond J. Safra Campus, 9190416 Jerusalem, Israel

## Abstract

The time-energy uncertainty relation (TEUR) $\Delta {\it{t}}\Delta {\it{E}}\,{\bf{ \ge }}\,{\textstyle{{\bf{1}} \over {\bf{2}}}}$ holds if the Hamiltonian is completely unknown, but can be violated otherwise; here we initiate a rigorous study describing when and to what extent such violations can occur. To this end, we propose a computational version of the TEUR (cTEUR), in which Δ*t* is replaced by the computational complexity of simulating the measurement. cTEUR violations are proved to occur if and only if the Hamiltonian can be fast forwarded (FF), namely, simulated for time *t* with complexity significantly smaller than *t*. Shor’s algorithm provides an example of exponential cTEUR violations; we show that so do commuting local Hamiltonians and quadratic fermion Hamiltonians. A general FF method is ruled out, but finding further examples, as well as experimental demonstrations, are left for future work. We discuss possible connections to sensing and quantum gravity. This work initiates a rigorous theory of efficiency versus accuracy in energy measurements using computational complexity language.

## Abstract (web summary)

A complete theory of when and to what extent time-energy uncertainty relation violations can occur is lacking. Here, the authors set the ground for a rigorous theory to investigate this possibility.

## Introduction

In quantum mechanics, the position-momentum uncertainty principle ${\rm{\Delta }}x \cdot {\rm{\Delta }}p \ge {\textstyle{1 \over 2}}$ can be proven from the properties of the Fourier transform for conjugate variables^1^. In the early years of quantum mechanics, energy and time were believed to be related similarly, by a so-called time-energy uncertainty relation (TEUR), though a similar proof does not apply since time is not an operator. Several alternative formulations of the TEUR were studied^2–5^. Here we consider the TEUR misconception^6,7^ stating that the duration Δ*t* of an energy measurement of an eigenstate $\left| {\psi _E} \right\rangle$ of a Hamiltonian *H* (referred to as the “input” Hamiltonian), is related to the standard deviation of that measurement Δ*E*, by

1 $${\rm{\Delta }}E \cdot {\rm{\Delta }}t \ge \frac{1}{2}.$$

Aharonov et al.^8^, proved that the TEUR (with a slightly different error quantification, see methods section) holds whenever the Hamiltonian *H* is completely unknown, namely, the experimentalist can only turn *H* on and off as if it resides in some black-box. Though not stated this way, the proof of^8^ holds also when only the eigenvalues of *H* are unknown. To the best of our understanding, the many recent experiments (e.g., refs. ^9–18^) demonstrating improved trade-offs between measurement resources and accuracy, all fall within this model, and as expected none exhibit a TEUR violation.

However, the TEUR is not a principle of Nature. Already in 1961, Aharonov and Bohm^19^ gave an example in which Eq. (1) can be violated to an arbitrary extent. They described a non-relativistic scattering experiment to measure the energy of a free particle, where the measurement’s accuracy depends on the time integral of the interaction *H*_meas._ between the system and the measurement device (importantly, *H*_meas._ is not equal to the input Hamiltonian *H*). By increasing the interaction strength by a factor *c* > 1 and applying $H_{{\mathrm{meas}}.}^\prime = cH_{{\mathrm{meas}}{\mathrm{.}}}$, the measurement duration becomes Δ*t*′ = Δ*t*/*c*, providing an arbitrarily large TEUR violation. A similar violation^8^ occurs when measuring the energy of a spin-1/2 particle in a known magnetic field by arbitrarily increasing the gradient of the magnetic field in a Stern-Gerlach experiment.

A complete theory of when and to what extent such TEUR violations can occur is missing. By^8^, such violations can only occur in the non-black-box setting, when there exists partial knowledge about the Hamiltonian. In this more general situation, various manipulations on the Hamiltonian can be conducted during the measurement and time duration of measurement can be traded with various other resources. As we discuss below, in such cases the TEUR no longer seems to correctly capture the question of resource-accuracy trade-off.

Our main contribution is in setting the grounds for a rigorous theory of TEUR violations in the general (not necessarily black-box) regime. To this end, we make use of the language of quantum computational complexity. We formulate below a modified, modern version of the TEUR, called the computational TEUR (cTEUR), in which Δ*t* is replaced by the computational complexity of the measurement process. This allows us to handle the full range of possible manipulations that can be applied during the energy measurement, ranging from simple manipulations as in ref. ^19^ to, ultimately, a full-fledged quantum computer aiding the measurement process. We argue that the computational complexity of the measurement correctly quantifies in all possible cases the total physical resources required to conduct the measurement. Here, by computational complexity of the measurement process, we mean the computational complexity of simulating the measurement process on a quantum computer.

Armed with the cTEUR, we turn to filling in some details in what seems to be an intricate emerging picture of possible cTEUR violations. First, we show that while completely unknown Hamiltonians obey the cTEUR, completely known Hamiltonians can lead to arbitrary violations of the cTEUR. Shor’s algorithm provides an intriguing example in which exponential violations are possible. Our main technical result is proving an equivalence between the ability to exponentially FF a Hamiltonian and measuring the energy of its eigenstates to within exponential accuracy. We show that two well studied classes of physical Hamiltonians can be FF: commuting local Hamiltonians and quadratic fermion Hamiltonians. We then show that not all physically realizable Hamiltonians can be FF (unless a strongly believed computational complexity conjecture is false). Finally, we discuss the relation of our work to metrology and sensing, as well as to recent ideas in the research of quantum gravity.

## Results

### The exact statement of the computational TEUR

Before defining the cTEUR, let us first clarify why the TEUR seems less suitable in a non-black-box setup such as in the Aharonov–Bohm example^19^. Note that the interaction strength (or the norm of the Hamiltonian) is not taken into account in the TEUR. In ref. ^19^, this “free” resource can thus replace time duration to achieve arbitrary violations of the TEUR. Time duration can also be traded with another resource. The spectral decomposition of the unitary evolution induced by the measurement Hamiltonian gives

2 $$e^{ - iH_{{\mathrm{meas}}{\mathrm{.}}}{\rm{\Delta }}t} = \mathop {\sum}\limits_j {\kern 1pt} e^{ - i\varepsilon _j{\rm{\Delta }}t}{\kern 1pt} \left| {\varepsilon _j} \right\rangle \left\langle {{\it{\varepsilon }}_j} \right|,$$

where *ε*_*j*_ are eigenstates of *H*_meas._. Evolving according to $H_{{\mathrm{meas}}.}^{{\prime\prime}} = \mathop {\sum}\nolimits_j {\kern 1pt} \left( {\varepsilon _j{\rm{\Delta }}t{\kern 1pt} \,{\mathrm{mod}}\,{\kern 1pt} 2\pi } \right)\,\left| {\varepsilon _j} \right\rangle \left\langle {\varepsilon _j} \right|$ for one time unit achieves the same unitary transformation as applying *H*_meas._ for time Δ*t*. Both the norm of the new Hamiltonian $H_{{\mathrm{meas}}.}^{\prime\prime}$ and the measurement’s duration (1 time unit) are now bounded, and yet arbitrarily good accuracy is achieved; the resource that is now being “freely” used is computational complexity. In order to apply $H_{{\mathrm{meas}}.}^{\prime\prime}$, one needs to diagonalize the original Hamiltonian and compute its eigenvalues to extremely high precision. What is revealed by the above discussion is that when manipulations can be done while performing the energy measurement, such as increasing the norm, using different measurement Hamiltonians, etc., this can lead to strong violations of the TEUR. Nevertheless the resources invested in the measurement have not decreased but were just interchanged with others!

Extending the intuition of resource counting in high-precision measurements (e.g. ref. ^20,21^), we argue that the “correct” notion that we would like to capture in the TEUR is not the time duration but the totality of physical resources one is required to invest in a measurement. The underpinnings of the area of quantum computation (see ref. ^22^) tell us exactly what is the right quantity to look at when counting resources: the computational complexity of the measurement, namely, the size of the quantum circuit simulating the process of the measurement, where size is measured by the number of two-qubit quantum gates^23^.

In order to state the computational TEUR (cTEUR), we need to clarify how we model an energy measurement. We use unitary implementations of energy measurements (called here “unitary energy measurements”), which entangle the input eigenstate *ψ*_*E*_ to a measurement device consisting of display and work registers, as follows:

3 $$U_{{\mathrm{meas}}{\mathrm{.}}}\,\left| {\psi _E} \right\rangle \, \left| {0,0} \right\rangle = \mathop {\sum}\limits_{E\prime ,E{\prime\prime} } {\kern 1pt} a_{E,E\prime ,E{\prime\prime} }{\kern 1pt} \left| {\psi _{E\prime }} \right\rangle \left| {E{\prime\prime} } \right\rangle \left| {\theta (E,E\prime ,E{\prime\prime} )} \right\rangle .$$

Measuring the second register in the computational basis gives the measurement outcome *E*′′. This unitary is in fact a quantum algorithm (comprised of local quantum gates^23^). Following ref. ^8^, we allow the circuit comprising *U*_meas._ also to apply the input Hamiltonian as a black-box for time *t*, namely to apply the operator *e* ^−*iHt*^ on any choice of a subsystem (this can be applied many times, where in between the applications we can have quantum gates). The complexity of this measurement process, denoted ${\cal T}(n)$, is the sum of two components: the first is the number of quantum gates utilized, the second is the total time the Hamiltonian was applied, divided by some canonical time unit *τ*_0_, where *τ*_0_ is the application time of a single quantum gate. We stress that classical pre- and post- processing should also be incorporated into the unitary energy measurement. The motivation for this definition is the assumption (by the quantum-complexity Church–Turing thesis^24,25^), that any quantum measurement process using the input Hamiltonian as a black-box, can be simulated by such a unitary energy measurement with at most polynomial overhead.

*Hypothesis 1* (computational TEUR (cTEUR)): A unitary energy measurement of an eigenstate of an *n*-qubit Hamiltonian *H*, with accuracy error *δE* satisfies

4 $$\delta E \cdot {\cal T}(n) \in {\mathrm{\Omega }}\left( {\frac{1}{{{\mathrm{poly}}(n)}}} \right).$$

*δE* (which replaces the standard deviation in Eq. (1)) is the accuracy error, namely the difference between the correct eigenvalue *E* and the measurement outcome *E*′′. Of course, accuracy is only guaranteed with some probability *η*, which we call confidence. We assume here *η* > 2/3. The notation Ω(1/poly(*n*)) means that $\delta E \cdot {\cal T}(n)$ is asymptotically larger than some function that is inverse polynomial in *n*. Ω(1/poly(*n*)) replaces the constant in the RHS of Eq. (1), to make the definition independent of the computational model, since ${\cal T}(n)$ may gain polynomial factors when translating from one model to another. The Ω notation implies that units of *E* are not important. Partial or full information about the Hamiltonian can be encoded into the unitary energy measurement; see Methods section for further details on the definition of the cTEUR. We thus arrive at a proposition which is rigorously defined and can be systematically studied.

As to the connection to TEUR, violating the cTEUR is strictly harder. First, violating the cTEUR implies violating the TEUR since the duration of time (measured in units of applying a single quantum gate) is always smaller than the total computational complexity. The other way around does not hold. An example is the case of the Aharonov–Bohm experiment^19^, which violates the TEUR but not necessarily the cTEUR because a straight-forward simulation of the measurement Hamiltonian *H*_meas._ of^19^ would result in computational complexity, which grows with the interaction strength (Supplementary Note 1).

### Violations of the cTEUR

We start by studying the two extreme cases of cTEUR violations. We first extend the proof of ref. ^8^ to show (Supplementary Note 3) that if *H* is completely unknown, or at least its eigenvalues are completely unknown, the cTEUR holds, just like the TEUR.

On the other hand, just like the TEUR, the cTEUR can be arbitrarily violated (though as discussed, not by the example of ref. ^19^). Such infinite violations follow immediately if we know everything there is to know about the Hamiltonian. We capture this by the notion of QC-solvable Hamiltonian. We say that an *n*-qubit Hamiltonian $H = \mathop {\sum}\nolimits_i {\kern 1pt} \lambda _i\left| {\psi _i} \right\rangle \left\langle {\psi _i} \right|$ is QC-solvable if it is diagonalized efficiently by a quantum computer (the transformation $\left| i \right\rangle \mapsto \left| {\psi _i} \right\rangle$ can be applied in poly(*n*) quantum complexity), and in addition, its eigenvalues can be efficiently found (the function $i\, \mapsto\, \lambda _i$ can be computed efficiently).

For a simple example, consider the following (clearly QC-solvable) Hamiltonian on *n* ${\textstyle{1 \over 2}}$-spins:

5 $$H = \mathop {\sum}\limits_{i = 0}^n {\kern 1pt} {\it{\sigma }}_i^z.$$

A measurement of the spins in the computational basis, followed by counting how many of the spins are in the state $\left| 1 \right\rangle$, would reveal the eigenvalue to infinite precision, namely, with *δE* = 0, and with confidence 1. This measurement can be implemented with linear computational complexity. It is straight forward to see that QC-solvable Hamiltonians allow for arbitrary violations of the cTEUR in much the same way (Supplementary Note 2 for definitions and proofs).

A very intriguing example which in fact triggered this work stems from Shor’s factoring algorithm^26^, which can be translated into an exponential violation of the cTEUR for a related class of Hamiltonians. In fact, this follows from the above since these Hamiltonians are QC-solvable (this is a rather simple exercise in quantum computation, using Shor’s algorithm). Nevertheless, the proof that these Hamiltonians violate the cTEUR contains the essential ingredients towards our main result in the next section, which provides an if-and-only-if condition for cTEUR even if the Hamiltonian is not QC-solvable.

Recall that Shor’s algorithm factorizes an *n*-bit number *N* by finding the order *r* of a randomly chosen *y* co-prime to *N*, namely the period of the sequence *y* ^0^, *y* ^1^, *y* ^2^ … modulo *N*. The algorithm uses the following unitary *U*_*N*,*y*_ acting on *n*-bit strings:

6 $$U_{N,y}\left| x \right\rangle = \left( {\begin{array}{*{20}{l}} {\left| {x \cdot y {\kern 1pt} \; {\mathrm{mod}}\;{\kern 1pt} N} \right\rangle } \hfill & {0 \le x < N} \hfill \\ {\left| x \right\rangle } \hfill & {{\mathrm{otherwise}}} \hfill \end{array}} \right.$$

*Theorem 1:* Let *N* be an *n*-bit integer and consider $H_{N,y} = U_{N,y} + U_{N,y}^{\mathrm{\dagger }}$ such that gcd(*y*, *N*) = 1. There exists a unitary energy measurement, which given any eigenstate of *H*_*N*,*y*_ has accuracy *δE* with confidence 2/3 such that:

7 $$\delta E \cdot {\cal T}(n) = O(2^{ - n}).$$

**Fig. 1:**
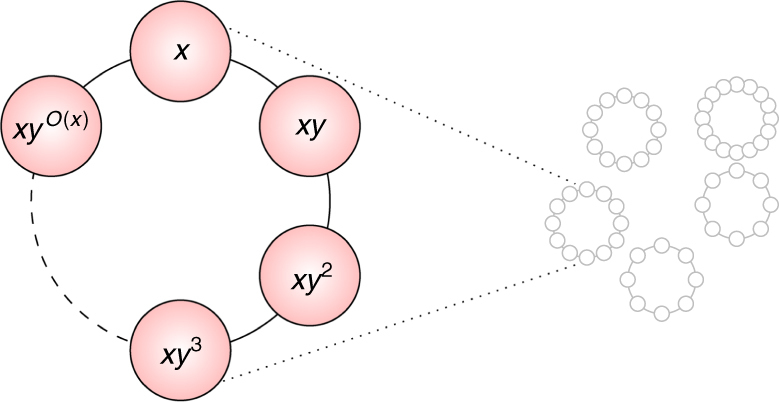
A physical intuition for *H*_*N*,*y*_. Vertices are the standard basis; edge (*u*, *v*) represents non-zero entry of *H*, namely 〈*u*|*H*_*N*,*y*_|*v*〉 = 1 and *v* = *uy* ^±1^ mod *N*. Multiplication by *y* mod *N* partitions the set {0, 1, … *N* − 1} into orbits whose (possibly exponential) sizes divide *r*, the order of *y*. *H*_*N*,*y*_ corresponds to a quantum walk^52^ along the cycles

Though *H*_*N*,*y*_ is not a local Hamiltonian, it is physically realizable (at least theoretically) as it can be simulated efficiently by a quantum computer (by refs. ^27,28^). See Fig. 1 for more on this Hamiltonian.

**Fig. 2:**
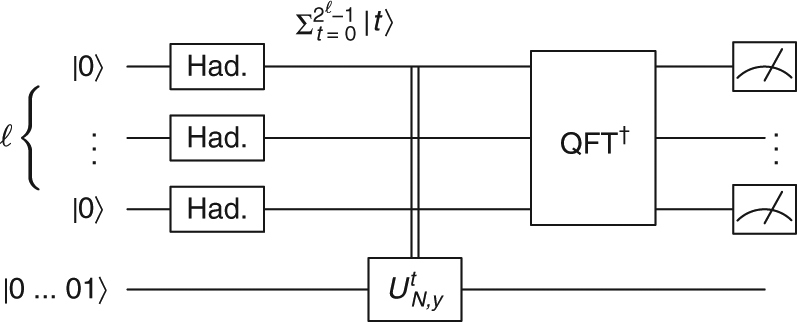
The familiar quantum circuit of Shor’s algorithm. If the input state is replaced by an eigenvector of *U*_*N*,*y*_, *ψ*_*φ*_ with eigenvalue *φ*, then the output is an exponentially accurate estimation of *φ*, implying an equally good estimation of the corresponding eigenvalue of *H*_*N*,*y*_. This reasoning holds with any unitary *e* ^−*iHt*^ instead of *U*_*N*,*y*_, hence efficient simulation of *e* ^−*iHt*^ for exponential *t* implies exponential cTEUR violation of *H*. This gives the seed of the proof of one direction of Theorem 2

**Fig. 3:**
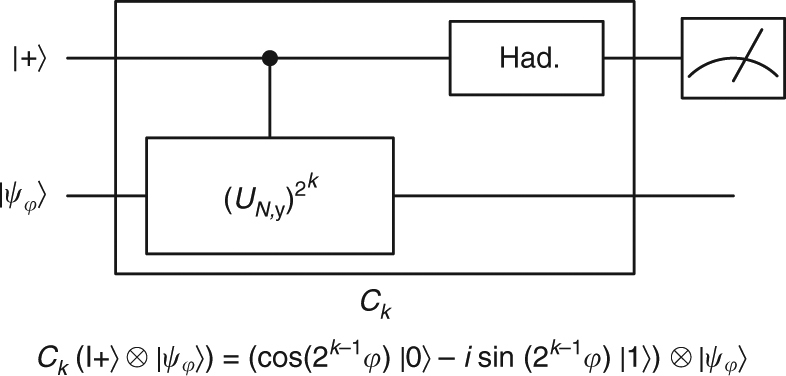
Proof of Theorem 1 by Kitaev’s phase estimation^29^. The circuit *C*_*k*_ is essentially a Mach-Zehnder interferometer, and $U_{N,y}\left| {\psi _\varphi } \right\rangle = e^{i\varphi }\left| {\psi _\varphi } \right\rangle$. The probability to measure 1 is $p_k = {\mathrm{sin}}^2{\kern 1pt} (2^{k - 1}\varphi )$. If (2*φ*/*π* mod 4) ∈ {0, 3}, ${p_0} < {\textstyle{1 \over 2}}$ otherwise ${p_0}  >{\textstyle{1 \over 2}}$. Similarly, the estimation of every *p*_*k*_ yields one bit of 2*φ*/*π*. The algorithm estimates each *p*_*k*_ by repeating the corresponding measurement poly(*n*) times, thus 1/exp(*n*) accuracy of *φ* is reached with total complexity poly(*n*): *C*_*k*_ is efficiently implemented even for *k* = poly(*n*) using modular exponentiation to implement exponential powers of *U*_*N*,*y*_ (Supplementary Note 4)

The proof is straight forward from Shor’s algorithm, in which eigenvalues of *U*_*N*,*y*_ are measured to exponential precision using the quantum phase estimation circuit^23^ (Figs. 2 and 3). One then uses the fact that *U*_*N*,*y*_ and *H*_*N*,*y*_ share eigenstates, and their eigenvalues are related by a simple transformation (Supplementary Note 4).

The above proof applies $U_{N,y}^t$ for exponentially large *t*, utilizing modular exponentiation, a poly(*n*) time classical algorithm to calculate exponential powers of *y* modulo *N*. Another way to view this is that the circuit efficiently simulates the Hamiltonian generating *U*_*N*,*y*_ for exponentially long times; this is an example of the notion of fast-forwarding to be defined in the next section, and already hints at its importance in precision measurements in general. Figure 3 describes an alternative proof of Theorem 1 based solely on fast forwarding and single qubit interference (Kitaev et al. phase-estimation algorithm^29^). Both proofs can be extended to prove our main Theorem in the next section.

### Fast forwarding and precision measurements

In our main technical result (Theorem 2), we provide an if-and-only-if condition for cTEUR violations. The result is stated using the notion of fast forwarding of Hamiltonians. A Hamiltonian *H* can be fast forwarded (FF) if the evolution with respect to *H*, to within time *t* (namely the unitary *e* ^−*iHt*^), can be simulated by a quantum computer in computational complexity (number of local gates) much smaller than *t* (similar notions were discussed elsewhere^30,31^). The definition is refined to allow some error *α* in the Hamiltonian simulation, as well as additional ancilla qubits that should be cleaned by the end of the simulation:

*Definition 1* (Fast forwarding a Hamiltonian (FF)). A normalized Hamiltonian *H* $\left( {\left\| H \right\| = 1} \right)$ acting on *n* qubits can be (*T*(*n*),*α*(*n*))-fast forwarded if for any *t* ≤ *T*, there exists a quantum circuit $\tilde U$ with poly(*n*) quantum gates, which acts on the *n* qubits and additional *c* = poly(*n*) ancilla qubits initialized to 0, s.t. for all *ψ*,

8 $$\left\| {\left( {e^{ - iHt} \otimes {\mathbb 1}_{2^c} - \tilde U} \right)\left| \psi \right\rangle \otimes \left| 0 \right\rangle } \right\| \le \alpha $$

We also need a more detailed version of the definition of unitary energy measurement. Here we allow usage of ancilla qubits (which we do not demand to be cleaned) and define a demolition parameter *β*, which quantifies imperfection in the measurement, and also limits how the input state changes.

*Definition 2* (*Super*-*Efficient energy measurements* (*SEEM*)). A normalized Hamiltonian *H* $\left( {\left\| H \right\| = 1} \right)$ acting on *n* qubits is (*η*, *δE*, *β*) − SEEM (super-efficient energy measurable) if there exist two unitaries $U_{{\mathrm{SEEM}}},\,\tilde U_{{\mathrm{SEEM}}}$, acting on the *n* qubits and on additional output/work qubits s.t.

1. *U*_SEEM_ is a measurement with accuracy *δE* and confidence *η*,
  9 $$U_{{\mathrm{SEEM}}}\left| {\psi _E} \right\rangle \left| {0,0} \right\rangle = \left| {\psi _E} \right\rangle {\kern 1pt} \mathop {\sum}\limits_{E\prime } {\kern 1pt} a_{E\prime }\left| {E\prime ,\theta (E\prime )} \right\rangle ,$$
  where *ψ*_*E*_ is an eigenstate, *E*′ is the measurement device’s output, and *θ*(*E*′) is the state of the ancilla qubits used in the measurement.
2. The complexity of implementing $\tilde U_{{\mathrm{SEEM}}}$ is polynomial in *n* and

10 $$\left\| {U_{{\mathrm{SEEM}}} - \tilde U_{{\mathrm{SEEM}}}} \right\| \le \beta .$$

By default, we will require that the demolition *β* is polynomially small in *n*; under this condition and assuming *η* > 2/3 (in fact, any constant *η* > 1/2 would do) the confidence parameter can be amplified, which means that with only polynomial overhead, the measurement can be improved to one with confidence exponentially close to 1 (See Lemma 1 in the SI).

*Theorem 2 [Main]:* For *n*, the number of qubits, the following two sets of Hamiltonians are equivalent:

1. FF_exp_: A normalized Hamiltonian *H* acting on *n* qubits is in FF_exp_ if there exists an exponentially growing function *T* = 2^Ω(*n*)^ s.t. *H* is (*T*, *α*)-FF for any *α* = *O*(1/poly(*n*)).
2. SEEM_exp_: A normalized Hamiltonian *H* acting on *n* qubits is in SEEM_exp_ if there exists a function *δE* = 2^−Ω(*n*)^ s.t. *H* is (*η*,*δE*, *β*)-SEEM for any *β* = *O*(1/poly(*n*)), *η* = 1 − *O*(1/poly(*n*)).

**Fig. 4:**
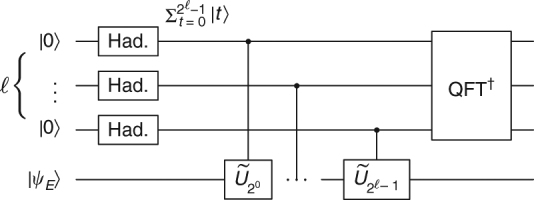
A circuit proving $H \in {\mathrm{FF}}_{{\mathrm{exp}}} \Rightarrow H \in {\mathrm{SEEM}}_{{\mathrm{exp}}}$. In this $\ell$-qubit phase estimation circuit, the gate $\tilde U_t$ is the *α*-approximation of *e* ^−*iHt*^; it is implemented efficiently for polynomial $\ell$ (or exponential *t*) if *H* ∈ FF_exp_. In that case, the circuit represents a SEEM. The FF error *α* accumulates additively, reducing confidence (*η*) and adding demolition to the energy measurement (*β*) (Supplementary Note 5)

**Fig. 5:**
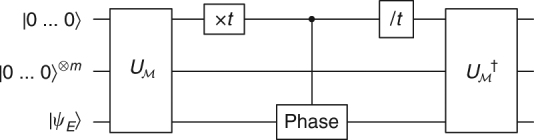
A circuit proving $H \in {\mathrm{SEEM}}_{{\mathrm{exp}}} \Rightarrow H \in {\mathrm{FF}}_{{\mathrm{exp}}}$. The gate $U_{\cal M}$ encapsulates *m* unitary energy measurements, the median of which is written on the topmost register (the median amplifies the confidence of the measurement). The state is then multiplied by a phase which equals the product of the median with *t*, after which $U_{\cal M}$ is undone to ensure a clean computation. Effectively, the circuit simulates *e* ^−*iHt*^ in polynomial complexity. Careful treatment is required for a measurement that perturbs *ψ*_*E*_, i.e., *β* > 0 (Supplementary Note 5)

The first direction, (FF implies SEEM), can be done by using phase estimation (Fig. 4) or Kitaev’s interference trick as in Fig. 3. For the other direction (SEEM to FF) see Fig. 5. Though the tools are quite standard, the error analysis is somewhat cumbersome since all parameters needed to be matched (Supplementary Note 5).

The attentive reader would notice that the proof described in Fig. 5 assumes a reversible measurement procedure. However, as mentioned, by the quantum-complexity Church–Turing thesis^24,25^, any physical process, including non-reversible processes, can be simulated by a quantum computer with a polynomial overhead. Hence under this thesis, our results apply to all energy measurements and not only to reversible ones.

### Fast-forwarding physical Hamiltonians

Using the equivalence provided by Theorem 2, we provide two new interesting classes of physical Hamiltonians, which are not known to be QC-solvable, yet can be exponentially FF (consequently, by Theorem 2, their energy eigenvalues can be efficiently measured to exponential precision): these are commuting local Hamiltonians, and quadratic fermion Hamiltonians (including Anderson localization^32^).

A commuting *k*-local Hamiltonian takes the form

11 $$H = \mathop {\sum}\limits_j {\kern 1pt} H_j,$$

where every term *H*_*j*_ acts non-trivially on at most on *k* qubits, and [*H*_*i*_, *H*_*j*_] = 0 for all *i*, *j*. When *k* is a constant (or even up to logarithmic), such Hamiltonians can be FF:

*Theorem 3*. If *H* is an *n*-qubit normalized commuting *k*-local Hamiltonian, with *k* = *O*(log(*n*)), then it can be (*T*,*α*)-fast forwarded for *T* = 2^*O*(*n*)^ and arbitrary exponentially small *α*.

Since the terms *H*_*j*_ commute, the problem reduces to FFing each term independently. This follows since the eigenvalues and eigenvectors of each local term can be efficiently calculated (even classically) with exponential accuracy^33,34^ (Supplementary Note 6).

Note that despite their simplicity, commuting local Hamiltonians generate highly non-trivial dynamics; they can efficiently generate distributions which are classically hard to sample from (under widely believed computational assumptions)^35,36^.

Similarly, quadratic fermion Hamiltonians can also be exponentially FF:

12 $$H = 	\mathop {\sum}\limits_{i,j}^m {\kern 1pt} A_{i,j}a_i^{\mathrm{\dagger }}a_j + \frac{1}{2}{\kern 1pt} \mathop {\sum}\limits_{ij} {\kern 1pt} B_{i,j}a_ia_j + \frac{1}{2}{\kern 1pt} \mathop {\sum}\limits_{i,j} {\kern 1pt} B_{j,i}^*a_i^{\mathrm{\dagger }}a_j^{\mathrm{\dagger }}\\ A = 	A^{\mathrm{\dagger }},B = B^{\mathrm{\dagger }}$$

where $a_i^{\mathrm{\dagger }},\,a_i$ are fermions creation and annihilation operators. Note that *n* indistinguishable fermions distributed over *m* = poly(*n*) modes are described by the Fock space of dimension $$\left( {\begin{array}{*{20}{c}} m \\ n \end{array}} \right)$$.

Assuming that we can physically implement any quadratic Hamiltonian, s.t. the error in each coefficient is at most inverse polynomial, we can thus prove:

*Theorem 4*. Let *H* be a quadratic Hamiltonian of *n* fermions with poly(*n*) modes. *H* can be (*T*,*α*)-fast forwarded with *T* = 2^*O*(*n*)^ and arbitrary inverse polynomial *α*.

The proof standardly uses the Bogoliubov transformation^37,38^ to “diagonalize” the traceless part of *H* and arrive at a free-fermion Hamiltonian (Supplementary Note 7). Extending to Bosons is left for future work.

### No generic fast forwarding

Perhaps any physically realistic Hamiltonian (one which is efficiently simulable by a quantum circuit) can be FF? A result of ref. ^39^ together with our Theorem 2 proves that this is impossible (assuming the common computational complexity assumption that BQP ≠ PSPACE (Supplementary Note 8 for definitions). Here we provide a simpler proof of this statement, which highlights the role of FF and SEEM (See Theorem 1 in SI).

The proof assumes by contradiction that any such Hamiltonian can be FF, and uses this to design a quantum polynomial time algorithm for the other end of this line (OEOTL) problem, which is known^40^ to be as hard as any problem in PSPACE. Such an algorithm contradicts the widely held assumption ${\mathrm{PSPACE}} \not\subseteq {\mathrm{BQP}}$.

*Definition 3* (OEOTL). Let *G* = (*V*, *E*) be a directed graph with 2^*n*^ vertices (indexed by *n* bits strings). *G* contains only directed paths, directed cycles, or isolated vertices. *G* is given by two polynomial size classical circuits: *S* (which computes the successor *S*(*u*) = *v* of a node *u* in *G*), and *P* (which computes the predecessor, *P*(*v*) = *u*)). We are promised that 0^*n*^ has no predecessor; the problem is to find the end of the line that starts with 0^*n*^.

**Fig. 6:**
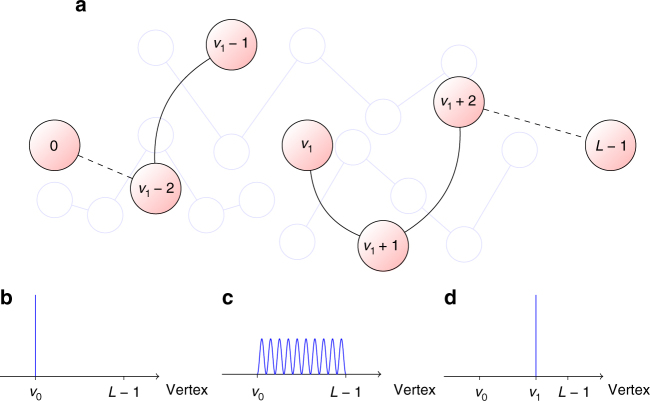
Solving OEOTL by FF. Let *H*_*G*_ be the adjacency matrix of the graph *G*, and denote by 0, 1, …, *L* − 1 the consecutive vertices of the line (*L* − 1 is the OEOTL). The panels show the probability distribution of the states in the first iteration of the algorithm. **b** The initial node *v*_0_ = 0. **c** *H*_0_ can be FF by assumption, and thus by theorem 2 we can SEEM with respect to *H*_0_ = *H*_*G*_, reaching approximately an eigenstate. **d** Measuring in the computational basis yields *v*_1_. Eigenstates are symmetric around the middle of the line, thus *v*_1_ is often found past the middle (a “successful iteration”). We iterate with $H_1 = H_0 - \left| {v_1} \right\rangle \left\langle {v_1 - 1} \right| - \left| {v_1 - 1} \right\rangle \left\langle {v_1} \right|$ to prevent recession (see **a** for the illustration of *H*_1_). We prove by standard argument from probability theory *n* of 100*n* iterations are successful with high probability, leaving the remaining path small enough for brute-force search to work. Here we assumed SEEM with demolition *β* = 0, but it’s not difficult to correct for small *β* (Supplementary Note 8)

A sketch for the algorithm is given in Fig. 6.

## Discussion

A fundamental question remains: What is the true physical reason that a system admits FF (or equivalently, SEEM), which systems allow it and to what extent? The straight-forward way to measure the energy of a given Hamiltonian is to apply it as a black-box; hence, one would expect the TEUR (and thus also the cTEUR) to hold in most physical experiments. However, should we expect a typical Hamiltonian to exponentially violate the cTEUR if we do allow non-black access? We suspect that most Hamiltonians do not allow such violations. Proving this would clarify the picture of possible cTEUR violations. One way toward a proof is to try to mimic our no-general-FF theorem 1 for a randomly chosen local Hamiltonian; perhaps this can be done by showing that a randomly chosen Hamiltonian is computationally universal (as in ref. ^41^); In fact, a stronger notion is needed, where the error when simulating polynomial quantum circuit is exponentially small. An intriguing question is whether many-body localized systems^42^, cousins of commuting local Hamiltonians, belong to this “fortunate” class of Hamiltonians allowing FF.

Two notions of “fully understanding” a Hamiltonian should not be confused. Having full information about the Hamiltonian means that we know all the parameters describing *H* (as in Theorems 1,3,4,1). This can be mathematically described as having an efficient quantum circuit simulating the Hamiltonian for unit time—which certainly does not imply FF (Theorem 1). On the other hand, we’ve shown that the Hamiltonian being QC-solvable (which is a much stronger notion of fully understanding the Hamiltonian) does imply FF. One may ask: is full information about the Hamiltonian needed to achieve FF? All our FF examples do make use of the exact Hamiltonian description (Theorems 3 and 4), but ref. ^8^ and theorem 7 do not rule out FF for partially known Hamiltonians. It is conceivable that in certain cases, one can use partial knowledge about the Hamiltonian in conjunction with quantum computational techniques to go beyond current super-sensitivity results^43,44^ bounded by the Heisenberg limit (see ref. ^45^). Additional connections of this work to metrology and sensing are discussed in Supplementary Note 9.

We believe that this work poses an important first step towards a rigorous theory of the possibilities of TEUR violations, and opens the exciting possibility that for certain Hamiltonians, efficient and extremely accurate energy measurements can be achieved using quantum computing techniques; these may be realizable even before full-fledged quantum computers exist.

## Methods

### Further details about the definition of cTEUR

We provide some missing details in the definition of the cTEUR. First, the confidence *η* is defined as follows.

*Definition 4* (*η*-accuracy): A unitary energy measurement as in Eq. (3) is said to have accuracy *δE* with confidence *η* (we say it is a measurement of *η*-accuracy *δE*) if given an eigenstate with energy *E*, the measurement outcome *E*′ satisfies

13 $$\mathop {{{\mathrm{Pr}}}}\limits_{\,\,\,\,\,E\prime } {\kern 1pt} \left( {\left| {E - E\prime } \right| \le \delta E} \right) \ge \eta .$$

We usually set *η* = 2/3. We note that any constant *η* > 1/2 can be amplified to become close to 1, assuming that the demolition parameter *β* is polynomially small. To see how this amplification is done, recall from definition 2 that polynomially small *β* implies that the perturbation of an eigenstate of *H* by the measurement is polynomially small. A small perturbation allows us to repeat the measurement *m* = poly(*n*) times, and calculate the median of the measurements outcome. The median would be within *δE* from *E* with probability, which approaches 1 exponentially fast in *m*, the number of repetitions (see the Confidence Amplification lemma, Lemma 1 below). The resulting unitary energy measurement would have the same *δE*, the confidence would be exponentially close to 1 and the demolition parameter would deteriorate by a factor of *m*.

We further explain the asymptotic notation Ω in the proposition. This notation is defined as follows: A function *f*(*n*) is said to be ∈ Ω(*g*(*n*)) if asymptotically it is larger than *cg*(*n*) for some non-negative constant *c*, namely, there exists a constant *c* > 0, and a natural number *n*_0_ s.t. *f*(*n*) > *cg*(*n*) for any *n* > *n*_0_. Hence, if $\delta E \cdot {\cal T}(n) \in \frac{1}{{{\mathrm{poly}}(n)}}$, this means that there exists an inverse polynomial function, which starting from some large enough *n*, bounds the product $\delta E \cdot {\cal T}(n)$ from below.

To be completely rigorous, we note that the cTEUR proposition should consider a family of Hamiltonians $\{ H_n\} _{n = 1}^\infty$ and a family of unitary energy measurements $\{ U_n\} _{n = 1}^\infty$, with increasing number of qubits. This is left implicit in this article. As is common in computational complexity, $\{ U_n\} _{n = 1}^\infty$ are assumed to be designed by a poly(*n*) classical algorithm whose input is *n*, and which may depend on any (possibly partial) information we have about the Hamiltonian.

We use the accuracy error as our error model because it conveniently translates to a unitary error in fast-forwarding (Theorem 2), but how does accuracy error compare to the standard deviation used in the TEUR?

Note that requiring the measurement to have accuracy error *δE* with confidence *η* is a slightly weaker requirement than the common requirement that the standard deviation is *δE*. In particular, when the standard deviation is specified, it is assumed implicitly that the expectation of the outcome is the correct value *E*. However, the expectation of the outcome *E*′ of a measurement of accuracy *δE* and confidence 2/3, might be arbitrarily far from *E*, namely, *δE* cannot give an upper bound on Δ*E* without further assumptions. A weak assumption suffices though. Assuming that $\left\| H \right\|$ is at most exponential, one can prove that ${\rm{\Delta }}E \le \left( {\sqrt \eta \delta E + 2\sqrt {1 - \eta } \left\| H \right\|} \right)$, and since *η* can be amplified to be exponentially close to one, this bound is meaningful (Supplementary Note 5). Conversely, *δE* cannot be bounded from above by Δ*E* because the standard deviation of the measurement could be 0 but still its expectation can be far from the correct *E*.

### Proof sketch of Theorem 2 (main)

We give here an outline of the main steps in the proof of Theorem 2. The proof builds on two tools. The confidence amplification lemma gives efficient exponential confidence amplification of a low-demolition energy measurement, without increasing the demolition parameter *β* too much:

*Lemma 1 (Confidence amplification)*. Let ${\eta}  >{\textstyle{1 \over 2}}$, and let *H* be a Hamiltonian on *n* qubits, $\left\| H \right\| \le 1$, which is (*η*, *δE*, *β*) − SEEM. Then for any integer *m* ≥ 1, *H* is also $( {1 - e^{ - {\textstyle{m \over 2}}\left( {1 - {\textstyle{1 \over {2\eta }}}} \right)^{\!\!2}},\,\delta E,\,m\beta } )$–SEEM.

The following lemma allows increasing the *T* parameter of FF at the cost of degrading *α*:

*Lemma 2 (FF by concatenation)*. For any integer *κ* > 0, if a Hamiltonian is (*T*, *α*)-FF, it is also (*Tκ*, *ακ*)-FF.

These lemmas can be used as follows to derive the proof. To prove the first direction, ${\mathrm{FF}}_{{\mathrm{exp}}} \Rightarrow {\mathrm{SEEM}}_{{\mathrm{exp}}}$: we first apply the FF by concatenation lemma (Lemma 2), to improve the FF parameters; using this improved FF ability, we can apply the phase estimation circuit (Fig. 4) to achieve highly accurate energy measurement, and lastly the parameters are improved by confidence amplification (Lemma 1).

To prove the other direction, ${\mathrm{SEEM}}_{{\mathrm{exp}}} \Rightarrow {\mathrm{FF}}_{{\mathrm{exp}}}$: The idea is to estimate the energy using the SEEM unitarily, then apply the correct phase (energy multiplied by the desired time) based on the resulting estimated value of the energy, and then run the energy estimation backwards to erase any garbage, in order to derive the unitary corresponding to the application of the Hamiltonian for time *t*. Once again, the confidence amplification lemma (Lemma 1) is required in order to gain back the parameters which were degraded.

**Fig. 7:**
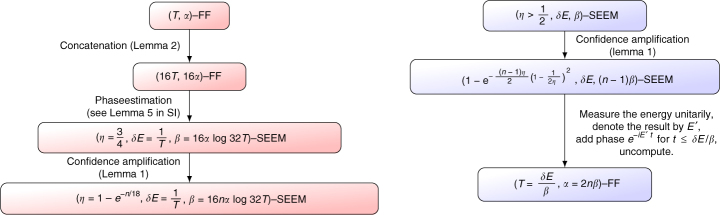
Sketch of the equivalence proof in both directions. The boxes indicate the guaranteed parameters, and the arrows are accompanied by the lemmas used to derive them

The details are not completely trivial due to the trade-off between the parameters, which all need to match up. The main steps are depicted in Fig. 7. See Supplementary Note 5 for full proofs of Theorem 2 and the Lemmas.

This proof, like the proof of Theorem 1 could be modified to rely on Kitaev’s phase estimation without making use of Fourier transform, which would give a more efficient computation from the point of view of making use of quantum computations versus classical ones.

We note that Theorem 2 can be extended to other functions *T*(*n*) and *δE* as a function of *n*. There seems to be, however, some inherent (constant) loss in parameters when moving between FF for time *T*, and SEEM with accuracy 1/*T*, at least in the way the above proof works; which is why Theorem 2 is stated using asymptotic functions.

### Relation of Theorem 1 to no-FF in other Hamiltonian models

We have discussed in the above the case of completely unknown Hamiltonians, as in ref. ^8^, as well as the general setting, which is the main subject of this article, in which we can have full information about the Hamiltonian (namely, we have a circuit for efficiently simulating the Hamiltonian for unit time) but the resources that are bounded are the computational complexity.

A different model, was studied in refs. ^28,30^ and is called the “Hamiltonian query model”. In this model, access to the matrix entries of the Hamiltonian is by queries to an oracle, which, given the index of a row, returns all non-zero elements in the row. This model seems less interesting from a physics perspective, however, there are interesting theoretical results which can be derived. In particular^30^ proved that there exist Hamiltonians, which require exponentially many queries in this model, in order to simulate their evolution to within exponential time. This can be viewed as a no-generic-FF theorem in this model.

We note that this theorem does not follow from Theorem 7, the cTEUR for unknown Hamiltonians (adapted from^8^, see Supplementary Note 3 for definition), though Theorem 7 together with our equivalence Theorem 2 implies a no-generic-FF for unknown Hamiltonians. The reason is that in the query model the Hamiltonian is not completely unknown, and possibly this additional information about the Hamiltonian can be used in order to achieve FF.

Summarizing the comparison between the three models, a Hamiltonian given as a black-box or one with unknown eigenvalues cannot be FF as this violates the TEUR/cTEUR for unknown Hamiltonians (This follows from the results of Aharonov et al.^8^ and our extension of it, Theorem 7 in Supplementary Note 3, together with our main Theorem 2). Adding information on the Hamiltonian when using the query model still won’t allow a general FF procedure due to query complexity bounds^30^. Theorem 1 is the corresponding theorem for the case of 2-sparse row computable Hamiltonians; Since we are no longer in the black-box model, nor even in the query model, we must condition the result on computational assumptions i.e., the widely believed assumption that PSPACE ≠ BQP (see Supplementary Note 8 for exact definition of these classes).

### Theorem 1 and a recent conjecture in quantum gravity

Theorem 1 is tightly related to a recent result by Aaronson and Susskind^46^, which was derived in the context of a conjecture in quantum gravity. This conjecture, due to Susskind^31^ connects the length of non-traversable wormholes to the computational complexity of approximating certain quantum states.

In this context, one is interested in a maximally entangled state, which evolves in time under the transformation:

14 $$\left| {\psi _t} \right\rangle = 2^{ - n{\mathrm{/}}2}{\kern 1pt} \mathop {\sum}\limits_{y = 1}^{2^n} {\kern 1pt} \left| y \right\rangle \otimes U^t\left| y \right\rangle ,$$

where *U* is a unitary related to the physical Hamiltonian in question (see ref. ^46^). Susskind^31^ proposed that the CFT dual of the length of non-traversable wormholes is equal to the quantum circuit complexity required to approximate $\left| {\psi _t} \right\rangle$. Aaronson and Susskind (manuscript in preperation; see ref. ^46^) do not handle the particular *U* of the CFT, but prove that there exists a unitary *U* such that the state in Eq. (14) is hard to approximate (more precisely, for some *t* < 2^*n*^, *ψ*_*t*_ with this *U* cannot be approximated efficiently) under a commonly believed computational assumption (${\mathrm{PSPACE}} \not\subseteq {\mathrm{PP/poly}}$). In their terminology, they show that there are no “shortcuts” to generating the state *ψ*_*t*_ for such a *U*. This closely ties with our no-generic FF Theorem 1, though it seems that their theorem does not directly imply Theorem 1. Note that if the Hamiltonian *H*_*S*_ generating the unitary *U*, s.t. $U = e^{ - iH_S}$, could be exponentially FF, the state complexity of *ψ*_*t*_ would by polynomial for *t* at most exponential. Thus, impossibility of FF of *H* follows from impossibility to generate *ψ*_*t*_ efficiently. The other way round might not hold—it is conceivable that FF is impossible, but the state *ψ*_*t*_ can be generated efficiently by a different way. This is why the computational assumption in Aaronson and Susskind’s result is stronger than ours, and involves the class PP and not BQP. The two other differences between the two theorems (Aaronson and Susskind work in the non-uniform setting, namely use PP/poly rather than PP, and consider approximation of the state to within a constant), depend on the setting and are less important.

An interesting question in this context is whether it is it possible to prove specifically that the above mentioned Hamiltonian *H*_*S*_ cannot be FF. Perhaps, this can be done using similar ideas to those mentioned in the discussion section.

### Quantum algorithms and fast-forwarding Hamiltonians

We have seen that the factoring algorithm can be interpreted as an efficient and exponentially accurate energy measurement utilizing fast forwarding. One can ask a conceptual question: is fast-forwarding Hamiltonians the true underlying source for all quantum algorithmic speed-ups? It turns out that in fact this is far from being the case. Indeed, like in Shor’s algorithm, the Abelian hidden subgroup problem (HSP) is solved^47,48^ by efficiently utilizing phase estimation to exponential accuracy, thus one can associate a Hamiltonian to the problem, and the quantum algorithm can be translated to a cTEUR violation in measuring the energies with respect to this Hamiltonian. We believe (though we have not worked out the details) that this is also the case for the recent extensions of Shor’s algorithm to finding unit groups of number fields^49,50^, which are also based on phase estimation of the eigenvalue of a unitary applied to exponential powers. However, to our current understanding, other than these few direct extensions of Shor’s algorithm, none of the other known quantum algorithmic speed-ups can be related to fast forwarding–not even quadratic fast forwarding (!). We note that some of these algorithms can be viewed an an energy measurement of a corresponding Hamiltonian, as we describe below, however, the quantum speed-up does not result from a FF of this Hamiltonian. We describe this in three interesting cases.

1. *The exponential speed-up of the quantum walk on two glued binary trees* ^51^: In this algorithm, an exponential quantum speed up is achieved by showing that a quantum algorithm can traverse a graph with exponentially many nodes, in polynomial time. The graph consists of two binary trees glued in their leaves. As shown in ref. ^51^, the glued trees problem is highly symmetric, and the search is limited to a subspace of dimension linear in the number of qubits. In addition ref. ^51^, show that the spectral gap of the Hamiltonian in that subspace is inverse polynomial.
  One can in fact view this process as an energy measurement, except not an accurate one. To see how continuous time quantum walks (CTQW)^52^ are related to energy measurements, consider the following analogy: In CTQW, a value *t* is chosen uniformly over [0, *T*] and the system is evolved by *e* ^−*iHt*^ and then measured. Almost equivalently, one can add to the state an ancilla register, initiated in the superposition over all values of time $\frac{1}{{\sqrt T }}{\kern 1pt} \mathop {\sum}\nolimits_{t = 0}^{T - 1} {\kern 1pt} \left| t \right\rangle$, and then apply the Hamiltonian on the state for a duration *t* conditioned that the value in the ancilla register is *t*, and finally discard the *t* register. This latter procedure is effectively a phase estimation (i.e., energy measurement), with the outcome traced out.
  However, the algorithm in ref. ^51^ only requires polynomial accuracy to perform this energy measurement, and in order to do this it simply applies the Hamiltonian for a polynomial amount of time, and does not utilize any fast-forwarding (equivalently, it does not violate the cTEUR).
2. *Grover’s quadratic algorithmic speed-up* ^53^: In Grover’s algorithm, an initial state $\left| s \right\rangle$, which is a uniform superposition over a search space of size *N* is rotated slowly to the marked state *ω*, and reaches its proximity after *O*(*N* ^−1/2^) applications of the iterator $U = \left( {{\mathbb 1} - 2\left| \omega \right\rangle \left\langle \omega \right|} \right)\,\left( {2\left| s \right\rangle \left\langle s \right| - {\mathbb 1}} \right)$. *U* may be written as:
  15 $$U = 	\left( {{\mathbb 1} - 2\left| \omega \right\rangle \left\langle \omega \right|} \right)\cdot \\ 	\left( {\frac{2}{N}( {\left( {N - 1} \right)\left| {s\prime } \right\rangle \left\langle {s\prime } \right| + \left| \omega \right\rangle \left\langle \omega \right| + \sqrt {N - 1} \left( {\left| {s\prime } \right\rangle \left\langle \omega \right| + \left| \omega \right\rangle \left\langle {s\prime } \right|} \right)} ) - {\mathbb 1}} \right),$$
  where $\left| s \right\rangle = \sqrt {(N - 1){\mathrm{/}}N} \left| {s\prime } \right\rangle + \sqrt {1{\mathrm{/}}N} \left| \omega \right\rangle$. The subspace spanned by *s*′, *ω* is invariant to *U*; by denoting $\left| \omega \right\rangle = \left| 0 \right\rangle$ and $\left| {s\prime } \right\rangle = \left| 1 \right\rangle$,
  16 $$\begin{array}{*{20}{l}} U \hfill & \hskip-8pt = \hfill &\hskip-7pt {\frac{1}{N}{\kern 1pt} \left( {\begin{array}{*{20}{c}} {N - 2} & { - 2\sqrt {N - 1} } \\ {2\sqrt {N - 1} } & {N - 2} \end{array}} \right)} \hfill \\ {} \hfill & \hskip-8pt = \hfill &\hskip-7pt {{\mathbb 1}{\kern 1pt} {\mathrm{cos}}{\kern 1pt} \left( {\frac{{2\sqrt {N - 1} }}{N}} \right) - i{\kern 1pt} {\mathrm{sin}}{\kern 1pt} \left( {\frac{{2\sqrt {N - 1} }}{N}} \right){\kern 1pt} \sigma ^y + O\left( {N^{ - 3{\mathrm{/}}2}} \right)} \hfill \\ {} \hfill & \hskip-8pt = \hfill &\hskip-7pt {e^{ - 2i\frac{{\sqrt {N - 1} }}{N}\sigma ^y} + O\left( {N^{ - 3{\mathrm{/}}2}} \right).} \hfill \end{array}$$
  Here we used the following:
  17 $$e^{i\varphi \sigma ^y} = {\mathbb 1}{\kern 1pt} {\mathrm{cos}}{\kern 1pt} (\varphi ) + i\sigma ^y{\kern 1pt} {\mathrm{sin}}{\kern 1pt} (\varphi )$$
  18 $$1 - \frac{2}{N} + O\left( {N^{ - 2}} \right) = {\mathrm{cos}}{\kern 1pt} \left( {\frac{{2\sqrt {N - 1} }}{N}} \right)$$
  19 $$\frac{{2\sqrt {N - 1} }}{N} + O\left( {N^{ - 3{\mathrm{/}}2}} \right) = {\mathrm{sin}}{\kern 1pt} \left( {\frac{{2\sqrt {N - 1} }}{N}} \right).$$
  Denote $H = 2\sigma ^y{\mathrm{/}}\sqrt N$; then *H* has eigenstates $\frac{1}{{\sqrt 2 }}\left( {\left| {s\prime } \right\rangle \pm i\left| \omega \right\rangle } \right)$, and additionally,
  20 $$\left\| {e^{ - iH} - U} \right\| = \left\| {e^{ - 2i\sigma ^y{\mathrm{/}}\sqrt N } - e^{ - 2i\sigma ^y\sqrt {N - 1} {\mathrm{/}}N} + O\left( {N^{ - 3{\mathrm{/}}2}} \right)} \right\| = O\left( {N^{ - 1}} \right).$$
  Measuring an eigenstate of *H* in the original standard basis returns *ω* with probability half. Thus, an algorithm equivalent to Grover’s is to apply an energy measurement of the state *s* with respect to the Hamiltonian *H*, with sufficient accuracy to arrive at a state close to an eigenstate, and then to measure in the original standard basis. Since the two eigenvalues differ by ≈*N* ^– 1/2^, it turns out that it suffices to perform a measurement with *η*-accuracy *N* ^−1/2^/10 for *η* = 1–10^−3^ to achieve probability at least 1/3 to measure *ω*. The exact argument follows from similar arguments to those in the proof of our no-general-FF Theorem 1, using claims 7 and 8 (see Supplementary Note 8). We omit the details. Thus, the quadratic speed-up is achieved by the mere fact that the accuracy required to separate the two eigenstates is of the order of $1{\mathrm{/}}\sqrt N$ and not 1/*N*.
3. *Exponentially fast solutions of linear equations* ^54,55^: The algorithm^54^ finds the state $\left| x \right\rangle = \mathop {\sum}\nolimits_i {\kern 1pt} x_i\left| i \right\rangle$ for *x* that solves the equation *Ax* = *b*. The matrix *A* is an *N* × *N* Hermitian *s*-row computable matrix, namely every row in *A* has at most *s* non-zero elements, and there exists an efficient algorithm recieving a row number as input, and outputing the position and values of these non-zero elements. The vector *b* is given as a state: $\left| b \right\rangle = \mathop {\sum}\nolimits_i {\kern 1pt} b_i\left| i \right\rangle$. The time complexity of the algorithm is $O({\mathrm{poly}}({\mathrm{log}}(N)),\kappa ,1{\mathrm{/}}\epsilon )$, where *κ* is the condition number of *A*, i.e., the ratio between the largest and smallest eigenvalues of *A*, and $\epsilon$ is the additive error of |*x*〉 allowed. The heart of the algorithm is a phase estimation of the unitary matrix *e* ^*iA*^ applied to the state |*b*〉. The Hamiltonian simulation procedures used to simulate *e* ^−*iAt*^ in ref. ^54,55^ apply for any *A*, thus both require at least linear computational complexity in *t*. If it weren’t so, one could violate cTEUR for unknown Hamiltonians—but this is, as we know, impossible (See Theorem 7 in SI). Hence no fast forwarding is involved.

As for other famous quantum algorithmic speed-ups, these do not seem to have a sensible description in terms of energy measurements of associated Hamiltonians, so they also do not seem to be related to FF. In particular, Kuperberg’s sub-exponential algorithm for finding a hidden subgroup of the Dihedral group^56^ and BQP-complete Topological Quantum Field Theory (TQFT) based quantum algorithms^57–59^, do not seem to have a FF origin.

### Data availability

The data sharing not applicable to this article as no data sets were generated or analyzed during the current study.

## Electronic supplementary material

Supplementary Information
