# Supplementary material for: Fast-forwarding of Hamiltonians and exponentially precise measurements
Source: Nat Commun. 2017 Nov 17;8:1572. doi: 10.1038/s41467-017-01637-7 (PMC5691059; doi:10.1038/s41467-017-01637-7)
Supplement: Supplementary file 1 — Supplementary Information [file 41467_2017_1637_MOESM1_ESM.pdf]

Supplementary Note 1: cTEUR violation implies TEUR violation

The main motivation for the definition of a complexity version of the TEUR (cTEUR), is that unlike the TEUR, the cTEUR takes into account resource exchange, and therefore distills the fact that some knowledge of the Hamiltonian is the necessary condition for violations. The implication is that TEUR violations due to resource exchange are not necessarily violations of the cTEUR. However, violating the cTEUR does imply a violation of the TEUR.

We prove that cTEUR violates the TEUR for energy accuracy defined by mean-deviation [1] as well as standard deviation.

Definition 5. Mean deviation of an energy measurement of an eigenstate with eigenvalue E is defined as

$$\underline{\Delta E} = \sum_{E'} \Pr(E'|E)\,|E - E'| \tag{1}$$

The claim is as follows:

Claim 1. Violating the cTEUR implies violating the TEUR. Let H be an n qubits Hamiltonian with norm $\|H\| = O(2^n)$, A unitary energy measurement with confidence $\eta = 2/3$ and $\beta = o(1/\text{poly}(n))$ implemented in computational complexity $\mathcal{T}(n)$, s.t. $\delta E\mathcal{T}(n) = o(1/\text{poly}(n))$, namely, a cTEUR violation, implies $\underline{\Delta E}\Delta t < 1/2$ where Δt is the measurement duration and $\underline{\Delta E}$ is the the mean deviation of the outcome. Similarly, cTEUR violation implies $\Delta E\Delta t < 1/2$.

The following lemma provides tools for the error comparison:

Lemma 3 (δE vs ΔE and $\underline{\Delta E}$)**.** Consider the distribution of an energy measurement of an eigenstate of H with eigenvalue E, s.t. the expectation value is $\bar{E}$.

a. $\Delta E \leq \eta(\delta E)^2 + 4(1-\eta)\|H\|^2 + 3(\eta\delta E + 2(1-\eta)\|H\|)^2$

b. $\underline{\Delta E} \leq \eta\delta E + 2(1-\eta)\|H\|$

Proof.

a:

We start with the following three identities, which we use to show that if the confidence is high, the standard deviation can't be much larger than δE.

$$\begin{aligned}(\bar{E} - E)^2 &= \left(\sum_{E'} \Pr(E'|E)(E' - E)\right)^2 \\ &= \left(\sum_{E':|E'-E|\leq\delta E} \Pr(E'|E)(E' - E) + \sum_{E':|E'-E|>\delta E} \Pr(E'|E)(E' - E)\right)^2 \\ &\leq (\eta\delta E + (1-\eta)\cdot 2\|H\|)^2,\end{aligned} \tag{2}$$

$$\begin{aligned}\sum_{E'} \Pr(E'|E)(E' - E)^2 &= \sum_{E':|E'-E|\leq\delta E} \Pr(E'|E)(E' - E)^2 + \sum_{E':|E'-E|>\delta E} \Pr(E'|E)(E' - E)^2 \\ &\leq \eta(\delta E)^2 + (1-\eta)(2\|H\|)^2,\end{aligned} \tag{3}$$

and similarly,

$$\sum_{E'} \Pr(E'|E)\cdot|E' - E| \leq \eta\delta E + 2(1-\eta)\|H\|. \tag{4}$$

The proof is as follows

$$
\begin{aligned}
(\Delta E)^2 &= \sum_{E'} \Pr(E'|E)(E'-\bar{E})^2 = \sum_{E'} \Pr(E'|E)(E'-\bar{E}\pm E)^2 \\
&\le \sum_{E'} \Pr(E'|E)\left[(E'-E)^2 + (E-\bar{E})^2 + 2\left|(E'-E)(E-\bar{E})\right|\right] \\
&\le \eta(\delta E)^2 + 4(1-\eta)\|H\|^2 + (\eta\delta E + 2(1-\eta)\|H\|)^2 + 2(\eta\delta E + 2(1-\eta)\|H\|)(\eta\delta E + 2(1-\eta)\|H\|) \\
&= \eta(\delta E)^2 + 4(1-\eta)\|H\|^2 + 3(\eta\delta E + 2(1-\eta)\|H\|)^2
\end{aligned}
\tag{5}
$$

b:

$$
\begin{aligned}
\underline{\Delta E} &= \sum_{E'} \Pr(E'|E)\,|E'-E| = \sum_{E':|E'-E|\le\delta E} \Pr(E'|E)\,|E'-E| + \sum_{E':|E'-E|>\delta E} \Pr(E'|E)\,|E'-E| \\
&\le \eta\delta E + 2(1-\eta)\|H\|
\end{aligned}
\tag{6}
$$

□

We can now proceed to the proof of the claim:

Proof. We first amplify the confidence by repeating the measurements $m = \mathrm{poly}(n)$ times, using Lemma 1 (declared in the methods section), thus accuracy δE and with $\eta' = 1 - e^{-\frac{m}{2}\left(1-\frac{1}{2\eta}\right)^2}$ and $\beta' = m\beta$. Substituting η and the Hamiltonian norm in Lemma 3b we get $\underline{\Delta E} \in o(1/\mathrm{poly}(n)$. The complexity of the amplification (assuming using linear complexity median algorithm) is $O(m\mathcal{T}(n))$ and its time duration is at most $\tau_0 m\mathcal{T}(n)$ where τ_0 is the time to apply one gate. Finally,

$$
\Delta t\underline{\Delta E} \le \tau_0 m\mathcal{T}(n)\underline{\Delta E} \in o(1/\mathrm{poly}(n)) \tag{7}
$$

Hence $\Delta t\underline{\Delta E}$ is asymptotically smaller than any constant and the TEUR is violated.

The same proof applies if standard deviation (ΔE) is used instead of mean deviation, using Lemma 3a,

$$
\Delta t\Delta E \le \tau m\mathcal{T}(n)\Delta E \in o(1/\mathrm{poly}(n)) \tag{8}
$$

□

We now explain in detail why the other direction might not be true (namely, violating the TEUR does not imply violating cTEUR) using the example of Y. Aharonov and Bohm [2]. This example violates the TEUR, however, it does not seem to violate the cTEUR. In this example the violation of the TEUR is achieved by increasing the interaction strength of the measurement Hamiltonian by an arbitrary factor $c > 0$. We claim that this increase is reflected also in an increased computational complexity of the measurement: Let $U_{\mathrm{meas.}} = e^{-iH_{\mathrm{meas.}}}$ be the measurement implementation, and let $f(n)$ be the time complexity of simulating the Hamiltonian $H_{\mathrm{meas.}}$ for one time unit (i.e., implementing $U_{\mathrm{meas.}}$). The naive way to simulate $cH_{\mathrm{meas.}}$ for one time unit, in order to improve the accuracy by a factor of c, is to concatenate c copies of the circuit implementing $e^{-iH_{\mathrm{meas.}}}$. This yields a total time complexity $cf(n)$ - and the factor c cancels with the one we get for the improvement in accuracy. But perhaps there are more efficient ways to simulate $e^{-iH_{\mathrm{meas.}}}$ than the naive way? Theorem 5 ensures us that there exists a Hamiltonian $H_{\mathrm{meas.}}$ s.t. if c is exponential in n, implementing $e^{-iH_{\mathrm{meas.}}c}$ requires super polynomial resources unless BQP = PSPACE (BQP, PSPACE are defined in Supplementary Note 8).

Supplementary Note 2: cTEUR Violations for fully known Hamiltonians

We revisit the simple counter example to the cTEUR described by equation (5) in the main text; it provides an infinite violation of the cTEUR (as well as of the TEUR) using a simple Hamiltonian on n spins (or qubits). Let

$$
H = \sum_{i=0}^{n} \sigma_i^z. \tag{9}
$$

Given an eigenstate, which is a tensor product of the eigenstates of each of the σ^z's, a measurement of each of the spins in the eigenbasis of the Pauli σ^z, (the computational complexity of this measurement is $O(n)$) reveals the eigenvalue to infinite precision, namely, with $\delta E = 0$. The demolition error however might be very large since most eigenstates are superposition of computational basis states. To avoid demolition altogether, an alternative measurement can be performed efficiently, using standard quantum computation tricks: Add a register of $\log(n)$ qubits all initiated in the state 0, and apply the unitary version of the classical computation which computes $w(i)$, the number of 1's in the string i of the original system, and writes it down on the additional register. In other words, apply the unitary operator:

$$U|i\rangle|0^{\log n}\rangle = |i\rangle|w(i)\rangle \tag{10}$$

this can be done using n times poly$(\log n)$ gates [3]. Now measure the right register, which gives the correct energy with $\delta E = 0$.

We note that here we have assumed that a single spin measurement in the computational basis can be performed with no error. We claim that a similar statement holds also when errors are taken into account, but a full analysis with noise (of this and other claims in the article) is left for future work.

More generally, consider the n qubit Hamiltonian $H = \sum_i \lambda_i |\psi_i\rangle \langle\psi_i|$, and assume that it is QC-solvable, i.e., we have full knowledge of its eigenstates and eigenvalues in the following sense: the functions $|i\rangle \mapsto |\psi_i\rangle$ and $i \mapsto \lambda_i$ can be computed by a quantum computer in polynomial time in n. An infinite violation of the cTEUR can be achieved: One can first apply the unitary $U = \sum_i |i\rangle \langle\psi_i|$ on the state to be measured, use the function $i \mapsto \lambda_i$ to write the energy on an ancilla register, and measure the ancilla. Finally apply U^{-1} to derive the original state again without any deviation.

These infinite violations assume full knowledge of the eigenstates and eigenvalues of the Hamiltonian in the above sense.

Supplementary Note 3: cTEUR/TEUR for unknown Hamiltonians

We observe the TEUR for completely unknown Hamiltonians [1], in fact holds for a more general setting, when only the eigenvalues are unknown. Namely, H is taken from a set of Hamiltonians all of which have the same set of eigenvectors, but we know nothing about their eigenvalues. We proceed to convert the mean-deviation used in [1] to $2/3-$accuracy (definition 4). Finally we use the previous results to prove a cTEUR for Hamiltonians with unknown eigenvalues.

Theorem 5 (TEUR for Hamiltonians with unknown eigenvalues (adapted from [1]))**.** If the eigenvalues of a Hamiltonian acting on a system are unknown, then the mean deviation $\underline{\Delta E}$ (see definition 5) with which one can estimate the energy of an eigenstate with energy E in a time duration Δt obeys the constraint

$$\underline{\Delta E}\Delta t > 1/4. \tag{11}$$

Proof. The proof of TEUR for completely unknown Hamiltonians [1] holds for the case in which only eigenvalues are unknown. One might be worried however that by applying the Hamiltonian on several probes in parallel on entangled states as is done for example in the case of NOON states (see Supplementary Note 9), one might be able to bypass the bound achieved in [1]. However, it was shown [4] that given any measurement scheme which applies the Hamiltonian on several probes (registers) in parallel, one can apply standard quantum computation techniques of adding a register and swapping between registers, to arrive at an equivalent protocol which only applies the Hamiltonian on one probe (register) sequentially. Hence we take Δt to be the total time the Hamiltonian was applied. □

In terms of $2/3$-accuracy, Theorem 5 takes the following form:

Theorem 6. Let H be a Hamiltonian with unknown eigenvalues. The $2/3$-accuracy δE of measuring the energy of an eigenstate depends on the time the Hamiltonian was sampled Δt by

$$\delta E \Delta t \geq 1/3 \tag{12}$$

Proof. Assume by contradiction that there exists some family of Hamiltonians with unknown eigenvalues (but fixed eigenstates which are common to all), and also that there exists a given eigenstate and constants δE and Δt, s.t. one can perform an energy measurement of the eigenstate with $2/3$-accuracy δE, while

applying the Hamiltonian for Δt, and yet $\delta E \Delta t < \frac{1}{3}$. We will derive a contradiction by showing that this implies a protocol which is too strong, for the distinguishability problem studied in [1].

The distinguishability problem is defined as follows: Given access to a Hamiltonian by a black box, determine whether the Hamiltonian in the box is H_1 or $H_2 = H_1 + \varepsilon \mathbb{1}$ (it is promised that the Hamiltonian in the box is one of the two, and it is assumed that there are no computational bounds outside the box, and in particular, we can feed the box any eigenstate we want). Both Hamiltonians have an a-priory probability 1/2. Define the probability of error for a protocol for this task by:

$$P_{\text{err}} = \frac{1}{2}\left[\Pr(\text{output } 2|H_1) + \Pr(\text{output } 1|H_2)\right]. \tag{13}$$

Using our assumed energy measurement, we can derive a protocol for this distinguishability task between two Hamiltonians from the family: H_1 and $H_2 = H_1 + \varepsilon \mathbb{1}$, with $\varepsilon = {}^2\!/_{3\Delta t}$. Apply an energy measurement with ${}^2\!/_3$-accuracy $\delta E < {}^1\!/_{3\Delta t} = {}^\varepsilon\!/_2$, to an eigenstate of the Hamiltonians (which by assumption we can generate). We know the energy of the eigenstate is either E or $E + \varepsilon$. The procedure outputs H_1 if the measurement outcome is closer to E than to $E + \varepsilon$ and outputs H_2 otherwise. From the definition of ${}^2\!/_3$-accuracy, in this procedure,

$$P_{\text{err}} < 1/3. \tag{14}$$

However, one of the intermediate results on Hamiltonian distinguishability in [1] is the following:

Lemma 4 (H distinguishability, adapted from [1] section III.B)**.** Any algorithm solving the distinguishability problem defined above for distinguishing between H_1 and $H_2 = H_1 + \varepsilon \mathbb{1}$, while applying the Hamiltonian in the black box for a total time Δt, satisfies

$$P_{\text{err}} \geq \frac{1}{2}\left[1 - \sin\left(\frac{\varepsilon \Delta t}{2}\right)\right] \tag{15}$$

if $\varepsilon \Delta t < \pi$.

Combining this lemma and Supplementary Equation (14) we have

$$\frac{1}{3} > P_{\text{err}} \geq \frac{1}{2}\left[1 - \sin\left(\frac{1}{3}\right)\right], \tag{16}$$

which is a contradiction. □

It is straight forward to argue that theorem 6 implies that also the cTEUR holds for Hamiltonians with unknown eigenvalues:

Theorem 7 (cTEUR for unknown eigenvalues)**.** Let H be a Hamiltonian whose eigenvalues are unknown. A unitary energy measurement of the input state with respect to H with accuracy δE and confidence 2/3 implemented in complexity $\mathcal{T}(n)$ satisfies:

$$\delta E \cdot \mathcal{T}(n) \in \Omega(1). \tag{17}$$

Proof. The proof follows trivially from Theorem 6. The total time the Hamiltonian is sampled if Δt, and the definition $\mathcal{T}(n) \in \Omega(\Delta t)$. Hence by Theorem 6,

$$\mathcal{T}(n)\delta E \in \Omega(\Delta t \delta E) = \Omega(1) \tag{18}$$

□

The reason we can prove here $\Omega(1)$ rather than just $\Omega(1/\text{poly})$ is that the bound is only due to duration the Hamiltonian was sampled, so the exact computational model is irrelevant.

Supplementary Note 4: Proofs of Theorem 1 (Shor's algorithm violates cTEUR)

Shor's algorithm factorizes an n-bit number N by finding the order r of a randomly chosen y co-prime to N (i.e., $\gcd(y, N) = 1$), namely the period of the sequence $y^0, y^1, y^2 ...$ modulo N. To this end, the algorithm uses the following unitary $U_{N,y}$ acting on n bit strings:

$$U_{N,y}\left|x\right\rangle = \begin{cases} \left|x \cdot y \mod N\right\rangle & 0 \le x < N \\ \left|x\right\rangle & \text{otherwise} \end{cases} \tag{19}$$

Theorem 1. Consider $H_{N,y}$ such that $\gcd(y, N) = 1$, and where N is an n-bit integer. There exists an energy measurement, which given any eigenstate of $H_{N,y}$ has accuracy δE with confidence $2/3$ such that:

$$\delta E \cdot \mathcal{T}(n) = O(2^{-n}). \tag{20}$$

The measurement procedure is such that the given eigenstate remains intact.

Proof by standard phase estimation

To achieve efficient and exponentially accurate measurement of the eigenvalues of $H_{N,y}$, we use the fact that $H_{N,y}$ shares the same eigenvectors with $U_{N,y}$, and their eigenvalues are related in a simple way. To see this, recall the orbit-stabilizer theorem [5] which implies that $U_{N,y}$ partitions the set $\{0, 1, ...N-1\}$ into orbits, each one being the orbit of some representative element in the set, and that the size of each orbit divides r. Denote x_ℓ the representative of the ℓ^{th}orbit, $\mathcal{O}(x_\ell)$. Then the eigenstates of $U_{N,y}$ (and of $H_{N,y}$) are of the form

$$\left|\psi_{\ell,k_\ell}\right\rangle = \sum_{j=0}^{|\mathcal{O}(x_\ell)|-1} e^{\frac{2\pi i j k_\ell}{|\mathcal{O}(x_\ell)|}} \left|x_\ell \cdot y^j \bmod N\right\rangle \qquad \begin{matrix} \ell \in \{1, 2, ..., \#\text{orbits}\} \\ k_\ell \in \{0, 1, ..., |\mathcal{O}(x_\ell)| - 1\} \end{matrix} \tag{21}$$

The eigenvalue of ψ_{ℓ,k_ℓ} with respect to $U_{N,y}$ is $e^{i\varphi} = e^{\frac{2\pi i k_\ell}{|\mathcal{O}(x_\ell)|}}$. The eigenvalue with respect to $H_{N,y}$ is $E_{\ell,k_\ell} = 2\cos(\varphi)$. Estimating E_{ℓ,k_ℓ} to within exponential accuracy will be achieved by a measurement of φ to within exponential accuracy. Note that in physics, $H_{N,y}$ describes a tight binding model of several disjoint 1D lattices, each of them with periodical boundary conditions.

The estimation of the eigenvalues of $H_{N,y}$ is thus a standard exercise in quantum computation; for completeness we include the details below. Importantly, we notice that though it might seem that the eigenvectors and eigenvalues of the Hamiltonian are known here in advance, in fact they are not - because they depend on r, which is computationally not known. This will prove that these Hamiltonians exhibit an exponential violation of the cTEUR, proving Theorem 1.

We are given y and n, and a quantum register containing an eigenstate $|\varphi\rangle$ of $H_{N,y}$ with an unknown eigenvalue $2\cos(\varphi)$. Let us fix an accuracy parameter $q = \text{poly}(n)$. We devise a procedure to estimate φ to within $2\pi \cdot 2^{-q}$ with $2/3$ probability, using only polynomially in q (and thus in n) quantum gates. This will prove the theorem.

Since the eigenvector is also an eigenvector of $U_{N,y}$, of eigenvalue $e^{i\varphi}$, we can use phase estimation (see section 5.2 in [3]) with respect to $U_{N,y}$ to estimate φ, which is directly related to the eigenvalue $2\cos(\varphi)$ we need.

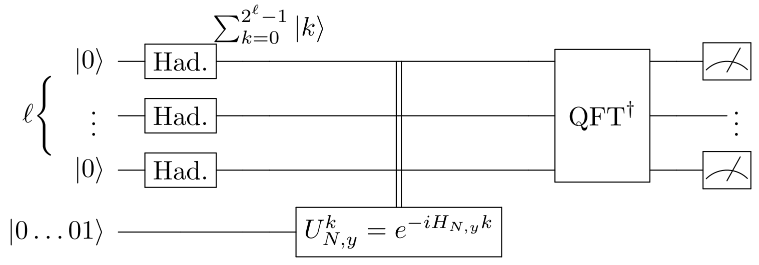

Supplementary Figure 1: An ℓ-bits phase estimation procedure used in Shor's algorithm. The phase is efficiently estimated to accuracy of $\frac{2\pi}{2^{\ell-2}}$ with confidence $\frac{1}{4}$.

The first step in phase estimation is to prepare an ancilla register in a superposition of values $0...L-1$, where $L = 2^\ell$, using ℓ Hadamard gates (see Supplementary Figure 1). We choose $\ell = q+6$. Then, $U^k_{N,y}$ is applied, conditioned that the value of the ℓ control bits is k in binary representation. Finally, the inverse of the quantum Fourier transform over $\mathbb{Z}_L$ is applied:

$$|k\rangle \stackrel{\mathrm{QFT}^\dagger}{\longrightarrow} \sum_{j=0}^{L-1} e^{-\frac{2\pi ijk}{L}} |j\rangle \tag{22}$$

This gives us the following sequence of implications:

$$\sum_{k=0}^{L-1} |\psi\rangle |k\rangle \rightarrow \sum_{k=0}^{L-1} U^k_{N,y} |\psi\rangle |k\rangle = \sum_{k=0}^{L-1} e^{ik\varphi} |\psi\rangle |k\rangle \rightarrow |\psi\rangle \sum_{j=0}^{L-1} |j\rangle \sum_{k=0}^{L-1} e^{i\left(k\varphi - \frac{2\pi jk}{L}\right)}. \tag{23}$$

Then the first ℓ qubits are measured and let m be the ℓ bit outcome. Then the output estimation of φ is $\varphi' = \frac{2\pi m}{2^\ell}$.

The following lemma is useful for evaluating the errors of phase estimation.

Lemma 5 (phase estimation confidence (adapted from 5.2.1 in [3]))**.** Let U be a unitary and $e^{i\varphi}$ an eigenvalue of U and let an eigenvector with this eigenvalue be given as input to the phase estimation procedure. Let m be the measurement outcome of an ℓ-qubits phase estimation circuit (see Supplementary Figure 1). For any $b+1 < \ell$,

$$\Pr\left(\left|\varphi - \frac{2\pi m}{2^\ell}\right| > \frac{2\pi}{2^b}\right) \leq \frac{1}{2(2^{\ell-b}-2)} \leq \frac{1}{2^{\ell-b}}. \tag{24}$$

Lemma 5 shows in our case, setting $b = q+4$, the probability the estimation φ' is $\pi \cdot 2^{-q-3}$ far from the value of φ is $\leq 1/4$. Since $E = 2\cos\varphi$, an accuracy $\delta\varphi$ in phase translates to accuracy $\delta E \leq 2\delta\varphi$. Hence the energy measurement outcome $E' = 2\cos\varphi'$ is 2^{-q}-far from the correct value E with probability at least $2/3$, as required.

The time complexity of the phase estimation is $O(n^3)$, which consists of preparing the superposition of the ancilla register ($O(n)$) quantum Fourier transform ($O(n^2)$) and the controlled application of U^k. The latter is done using the method of modular exponentiation. To compute $U^k_{N,y}$ one needs to multiply a given integer by y raised to the power k modulo N. To this end, the sequence $y^{2^1}, y^{2^2}, ..., y^{2^\ell} \bmod N$ is calculated classically using repeated squaring ($O(n)$ multiplications of integers written on $O(n)$ size registers yields $O(n^3)$ 2-gate operations), and then $O(n)$ ctrl-$(U_{N,y})^k$ gates are applied for a total time complexity of $O(n^3)$. Shor [6] improved the complexity to $O(n^2 \log n \log\log n)$ by using improved integer multiplication techniques.

Proof by Kitaev's phase estimation

A much simpler algorithm to measure the energy exists, more along the lines of Kitaev's original presentation of phase estimation [7]. Given an eigenstate ψ_E, then by adding a control qubit in the state $\frac{1}{\sqrt{2}}(|0\rangle + |1\rangle)$ in another register, and applying U^t conditioned on the control qubit being 1, the state of the control qubit becomes:

$$\frac{1}{\sqrt{2}}(|0\rangle + e^{-iEt} |1\rangle) \tag{25}$$

For each $t \in \{2^k\pi | k = 0, 1, ...\mathrm{poly}(n)\}$ we can generate polynomially many control qubits in this state. Measuring these qubits (for a fixed k) in the $\{|+\rangle, |-\rangle\}$ basis enables evaluating one bit of the energy, with exponentially good confidence in a similar way to evaluating the bias of a classical coin by taking the average over many trials.

Formally, the phase estimation algorithm is iterative, and is similar to binary search. In the jth iteration ($j \in \{1, 2, ...\mathrm{poly}(n)\}$) the phase estimated φ is assumed to be in the interval $[\varphi^j_{\min}, \varphi^j_{\max}]$, with $\Delta_j \triangleq \varphi^j_{\max} - \varphi^j_{\min} = \pi/t_j$ and $t_j = 2^{j-1}$. We generate $m = \mathrm{poly}(n)$ many control qubits in the state

$$\frac{1}{\sqrt{2}}(|0\rangle + e^{-i(\varphi - \varphi^j_{\min}) \cdot t_j} |1\rangle), \tag{26}$$

apply Hadamard on each one of these qubits and measure in the $\{|0\rangle, |1\rangle\}$ basis (See Supplementary Figure 2 for a schematic description of the circuit for one such control qubit). This can be done efficiently: the phase φt_j is added efficiently by modular exponentiation, and the known phase dependent on φ^j_{min} is added by using standard quantum computation techniques. The outcome 1 is achieved with probability

$$p_j = \sin^2((\varphi - \varphi^j_{\text{min}})t_j/2) = \sin^2\left(\frac{\varphi - \varphi^j_{\text{min}}}{\Delta_j} \cdot \frac{\pi}{2}\right) \tag{27}$$

Let $\widetilde{p}_j$ be the fraction of the measurements with 1 outcome, and let $\widetilde{\varphi}_j = \frac{2}{t_j}\arcsin(\sqrt{\widetilde{p}_j}) + \varphi^j_{\text{min}}$. It will serve as the current estimate of φ. For the $(j+1)^{\text{th}}$ iteration's interval (with length Δ_{j+1}), we choose

$$\varphi^{j+1}_{\text{min}} = \begin{cases} \varphi^j_{\text{min}} & \widetilde{\varphi} - \varphi^j_{\text{min}} \leq \frac{\Delta_j}{3} \quad (\widetilde{p}_j \leq \frac{1}{4}) \\ \varphi^j_{\text{min}} + \frac{\Delta_j}{4} & \frac{\Delta_j}{3} < \widetilde{\varphi} - \varphi^j_{\text{min}} \leq \frac{2\Delta_j}{3} \quad (\frac{1}{4} < \widetilde{p}_j \leq \frac{3}{4}) \\ \varphi^j_{\text{min}} + \frac{\Delta_j}{2} & \widetilde{\varphi} - \varphi^j_{\text{min}} > \frac{2\Delta_j}{3} \quad (\widetilde{p}_j > \frac{3}{4}) \end{cases} \tag{28}$$

and of course $\varphi^{j+1}_{\text{max}} = \varphi^{j+1}_{\text{min}} + \Delta_{j+1}$.

Assuming we chose the correct interval in every iteration, after ℓ iterations we know the phase with accuracy $\Delta_{\ell+1} = 2^{-\ell}\pi$.

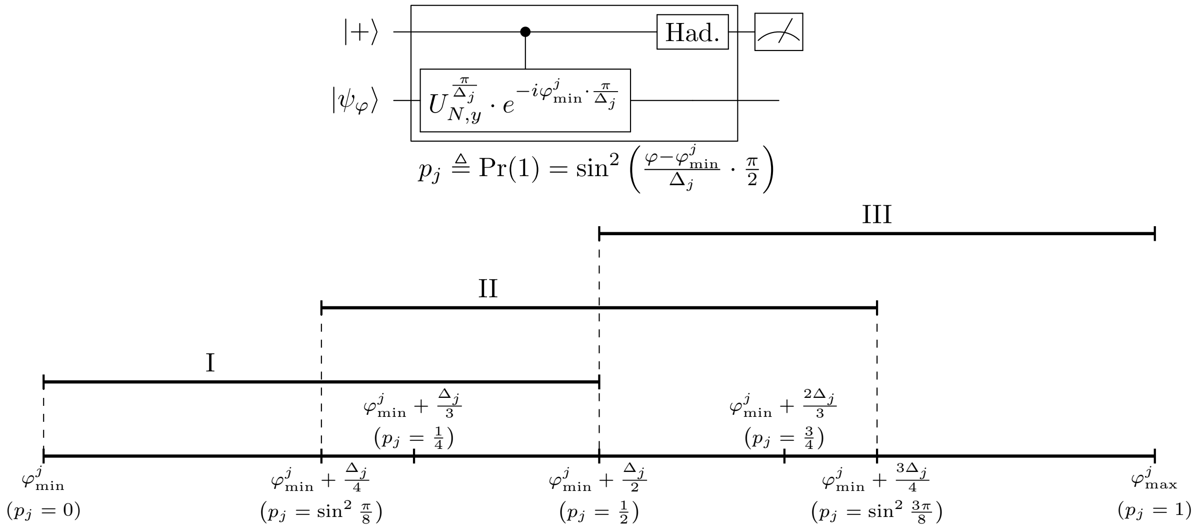

Supplementary Figure 2: Phase estimation without Fourier transform. Let ψ_φ be an eigenstate with (unknown) eigenvalue $e^{i\varphi}$. The algorithm is very similar to the binary search algorithm: initially φ is confined to the initial interval $[\varphi^1_{\text{min}}, \varphi^1_{\text{max}}]$ of size Δ_1, and in every iteration the interval is halved. Unlike binary search, here there are 3 ways to halve the interval (I,II,III). At each iteration, the circuit (top) is applied $m = \text{poly}(n)$ times, and $\widetilde{p}_j$ which denotes the ratio of 1s approximates the actual probability to measure 1, p_j which depends on φ. Then, according to $\widetilde{p}_j$, the next interval, I, II, or III is selected. After $\ell = \text{poly}(n)$ iterations the interval's size is $2^{-\ell}\Delta_1$.

Lemma 6. Let $e^{i\varphi}$ be the eigenvalue of the unitary $U_{N,y}$, and $\varphi \in [\varphi^1_{\text{min}}, \varphi^1_{\text{min}} + \pi]$ for a known φ^1_{min}. The phase estimation procedure described above, denoted $f(U_{N,y}, [\varphi^1_{\text{min}}, \varphi^1_{\text{min}} + \pi])$, finds φ with accuracy $2^{-\ell}\pi$ and with confidence greater than $1 - \ell e^{-m/160}$, where ℓ is the number of iterations, and m is the number of times the circuit in Supplementary Figure 2 is applied in each iteration.

Proof. In each iteration there are four scenarios where we choose the wrong interval for the next iteration. We use the Chernoff bound and its monotonicity to find a bound on the error probability in each scenario, and then pick the largest one.

$$\Pr(\widetilde{p}_j \geq \gamma) \leq e^{-\frac{mp_j}{3}(\frac{\gamma}{p_j}-1)^2} \qquad \gamma > p_j \tag{29}$$

$$\Pr(\widetilde{p}_j \leq \gamma) \leq e^{-\frac{mp_j}{2}(\frac{\gamma}{p_j}-1)^2} \qquad \gamma < p_j \tag{30}$$

1. The phase is in I$-$II (see Supplementary Figure 2), i.e., $\varphi \in [\varphi^j_{\min}, \varphi^j_{\min} + \frac{\Delta_j}{4})$, but we measured $\widetilde{\varphi}_j > \varphi^j_{\min} + \frac{\Delta_j}{3}$ thus picked intervals II or III:
$$\Pr\left[\widetilde{p}_j > \frac{1}{4} \cap p_j < \sin^2(\frac{\pi}{8})\right] \le e^{-(\frac{1}{4\sin^2(\frac{\pi}{8})}-1)^2 m \sin^2(\frac{\pi}{8})/3} = e^{-m(\sqrt{2}-1)/12\sqrt{2}} \tag{31}$$
(we used $\sin^2(\pi/8) = \frac{\sqrt{2}-1}{2\sqrt{2}}$)

2. The case symmetric to 1; φ is in III$-$II and intervals I or II were chosen:
$$\Pr\left[\widetilde{p}_j < \frac{3}{4} \cap p_j > \sin^2(\frac{3\pi}{8})\right] \le e^{-(\frac{3}{4\sin^2(\frac{3\pi}{8})}-1)^2 m \sin^2(\frac{3\pi}{8})/2} = e^{-m\frac{(1+\sqrt{2})^3}{8\sqrt{2}}} \tag{32}$$

3. The phase is in (I$\cap$II)$-$III, i.e., $\varphi \in [\varphi^j_{\min} + \frac{\Delta_j}{4}, \varphi^j_{\min} + \frac{\Delta_j}{2})$ but we measured $\widetilde{\varphi} > \varphi^j_{\min} + \frac{2\Delta_j}{3}$ thus picked interval III:
$$\Pr\left[\widetilde{p}_j > \frac{3}{4} \cap p_j \in \left[\sin^2(\frac{\pi}{8}), \frac{1}{2}\right)\right] \le e^{-\frac{m}{24}} \tag{33}$$

4. The case symmetric to 3; φ is in (III$\cap$II)$-$I, and interval I was chosen:
$$\Pr\left[\widetilde{p}_j < \frac{1}{4} \cap p_j \in \left(\frac{1}{2}, \sin^2(\frac{3\pi}{8})\right]\right] \le e^{-\frac{m}{16}} \tag{34}$$

All these errors are smaller than $e^{-m/160}$. The probability to be in the wrong interval after ℓ iterations follows from the union bound and is at most $\ell e^{-\frac{m}{160}}$. □

In general,we have no prior knowledge that φ is in some window of size π, and so it can be any value in $[0, 2\pi]$. Using $f(U_{N,y}, 0)$ on an unbounded φ creates an ambiguity: φ and its mirror value $2\pi - \varphi$ would create the same distribution of measurement outcomes in every iteration. The following algorithm solves the ambiguity by trying to run f on a range containing both values:

1. Let $\widetilde{\varphi} = f(U_{N,y}, [0, \pi])$
2. If $\left|\widetilde{\varphi} - \frac{\pi}{2}\right| > 2^{-\ell}\pi$,
   run f on $U_{N,y}$ in a range containing both $\widetilde{\varphi}$ and $2\pi - \widetilde{\varphi}$ (i.e. $[\frac{\pi}{2}, \frac{3\pi}{2}]$ or $[0, \frac{\pi}{2}] \cup [\frac{3\pi}{2}, 2\pi]$); return the result.
3. Else (Unable to determine whether φ and $2\pi - \varphi$ are in $[\frac{\pi}{2}, \frac{3\pi}{2}]$ or in $[0, \frac{\pi}{2}] \cup [\frac{3\pi}{2}, 2\pi]$)
   (a) Let $U'_{N,y} = e^{i\pi/4} U_{N,y}$
   (b) Let $\widetilde{\xi} = f(U'_{N,y}, [0, \pi])$
   (c) If $\left|\widetilde{\xi} - \frac{\pi}{2}\right| > 2^{-\ell}\pi$,
   run f on $U'_{N,y}$ in a range containing both $\widetilde{\xi}$ and $2\pi - \widetilde{\xi}$, (i.e. $[\frac{\pi}{2}, \frac{3\pi}{2}]$ or $[0, \frac{\pi}{2}] \cup [\frac{3\pi}{2}, 2\pi]$); return the result minus $\frac{\pi}{4}$.
   (d) Else, the algorithm fails.

If every time f is used, it finds the correct phase (or its mirror value) with accuracy $2^{-\ell}\pi$, then the algorithm successfully finds φ with accuracy $2^{-\ell}\pi$. f is called at most three times, therefore by Lemma 6 the algorithm above finds φ with accuracy $2^{-\ell}\pi$ and with confidence greater than $1 - 3\ell e^{-m/160}$.

Once φ is approximated for $U_{N,y}$, the corresponding energy under $H_{N,y}$ can be calculated by the relation $E = 2\cos(\varphi)$. The complexity of the algorithm is polynomial in the number of qubits therefore the proof of theorem 1 is completed.

Supplementary Note 5: Proof of theorem 2 (main)

Theorem 2. **[Main]** For n the number of qubits, the following two sets of Hamiltonians are equivalent:

1. $\mathsf{FF}_{\exp}$: A normalized Hamiltonian H acting on n qubits is in $\mathsf{FF}_{\exp}$ if there exists an exponentially growing function $T = 2^{\Omega(n)}$ s.t. H is (T, α)-FF for any $\alpha = O(1/\text{poly}(n))$.

2. $\mathsf{SEEM}_{\exp}$: A normalized Hamiltonian H acting on n qubits is in $\mathsf{SEEM}_{\exp}$ if there exists a function $\delta E = 2^{-\Omega(n)}$ s.t. H is $(\eta, \delta E, \beta)$-SEEM for any $\beta = O(1/\text{poly}(n)), \eta = 1 - O(1/\text{poly}(n))$.

The proof builds on two tools. The confidence amplification lemma (Lemma 1) gives efficient exponential confidence amplification of a low-demolition energy measurement, without increasing the demolition parameter β too much. The second tool is the FF by concatenation Lemma (Lemma 2) allows increasing the T parameter of FF at the cost of degrading α. We note that we do not know how to apply confidence amplification to $2^{-\Omega(n)}$ when β is not $O(1/\text{poly}(n))$. Theorem 2 leaves open the possibility that measurements with larger demolition are not equivalent to FF (this remains to be studied).

Lemma 1 (Confidence amplification)**.** Let $\eta > \frac{1}{2}$, and let H be a Hamiltonian on n qubits, $\|H\| \leq 1$, which is $(\eta, \delta E, \beta)-$SEEM. Then for any integer $m \geq 1$, H is also $(1 - e^{-\frac{m}{2}\left(1-\frac{1}{2\eta}\right)^2}, \delta E, m\beta)-$SEEM.

Proof. Consider m applications of the non-perturbing unitary energy measurement circuit U_{SEEM} with η-accuracy δE. The probability that the majority of these outputs are within δE of the correct value can be bounded by the Chernoff bound

$$\Pr(\text{majority of measurements outside the window } \delta E) \leq e^{-\frac{m}{2}\left(1-\frac{1}{2\eta}\right)^2} \tag{35}$$

Hence a median of the measurements is at distance $\leq \delta E$ from the correct energy value with confidence $1 - e^{-\frac{m}{2}\left(1-\frac{1}{2\eta}\right)^2}$. We define the new measurement circuit V_{SEEM} to first apply U_{SEEM} m different times, each time using a new ancilla register. Each such circuit writes E' on its ancilla register. V_{SEEM} then unitarily computes the median of these m outputs on an extra register. We know that had one of these outputs been measured, the probability that it is within δE from the correct value E is at least η. Since the measurements of those values mutually commute, are independent, and commute with the measurement of the median, we see that the median is within δE from E with probability at least $1 - e^{-\frac{m}{2}\left(1-\frac{1}{2\eta}\right)^2}$.

$\widetilde{V}_{\text{SEEM}}$ is defined by replacing U_{SEEM} m by $\widetilde{U}_{\text{SEEM}}$ in the above procedure. Since this is done m times we have

$$\|\widetilde{V}_{\text{SEEM}} - V_{\text{SEEM}}\| \leq m\|\widetilde{U}_{\text{SEEM}} - U_{\text{SEEM}}\| \leq m\beta. \tag{36}$$

□

The second tool allows increasing the T parameter of fast forwarding at the cost of degrading α.

Lemma 2 (FF by concatenation)**.** For any integer $\kappa > 0$, if a Hamiltonian is (T, α)-FF, it is also $(T\kappa, \alpha\kappa)$-FF.

Proof. The proof is by concatenation of κ instances of the fast-forwarding circuit; the bound of $\alpha\kappa$ is derived by a standard telescopic argument. □

To prove Theorem 2 we start by proving that fast forwarding implies super efficient energy measurements. After this we prove the other direction. The proof structure is in Supplementary Figure 3.

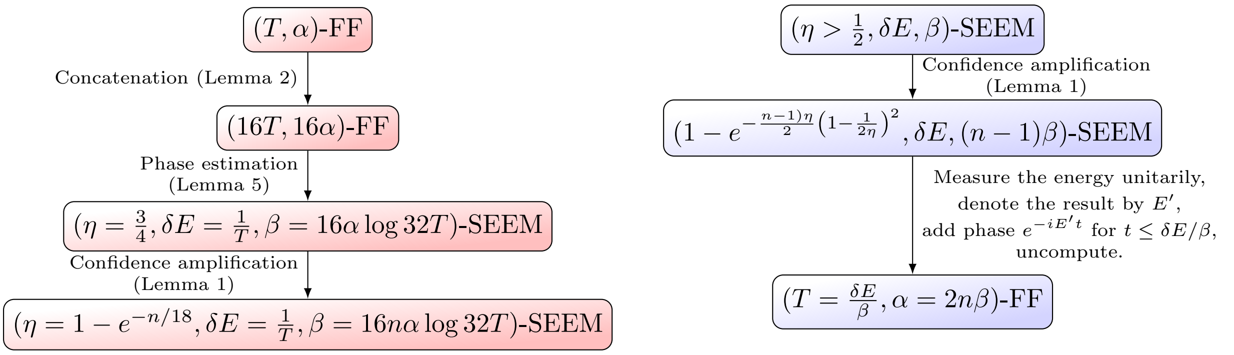

Supplementary Figure 3: Sketch of the equivalence proof in both directions. The boxes indicate the guaranteed parameters, and the arrows are accompanied by the lemmas used to derive them.

Claim 2. For $T = O(2^{\text{poly}(n)})$, if a normalized Hamiltonian on n qubits is (T, α)-FF, it is additionally $(1 - e^{-n/18}, \frac{1}{T}, 16n\alpha \log(32T))$-SEEM.

Proof. We start by using the concatenation lemma (Lemma 2) to claim the Hamiltonian is $(16T, 16\alpha)$-FF. Next we show that $(16T, 16\alpha)$-FF and $T = O(2^{\text{poly}(n)}) \Rightarrow (\frac{3}{4}, \frac{1}{T}, 16\alpha \log(32T))$-SEEM. The result then follows from the amplification lemma, Lemma 1 with $m = n$.

We use the assumption that fast forwarding of H is possible, to efficiently apply phase estimation with respect to the unitary $V = \exp(i(H + \mathbb{1}))$. V and H of course share eigenvectors, and an eigenvalue E of H corresponds to an eigenvalue $e^{i\varphi}$ for V for $\varphi = E+1$ (recall that $\|H\| \le 1$ so $0 \le \varphi = E+1 \le 2 \le 2\pi$).

Fix $\ell = \lfloor \log(32T) \rfloor$ to be the number of bits of φ estimated in the phase estimation procedure. The procedure requires conditional applications of $\left\{V^{2^k}\right\}_{k=0}^{\ell-1}$; This is done by implementing ℓ different instances of fast forwarding of H, e^{iHt}, with $t = 2^0, 2^1 \ldots 2^{\ell-1} \le 16T$.

Using Lemma 5, we get that the ℓ-bit phase estimation procedure estimates φ to within $\delta\varphi = \pi \cdot 2^{-(\ell-3)}$ with confidence 3/4. We get that the procedure provides an outcome which is within $\delta E = \delta\varphi = 4\pi \cdot 2^{-(\ell-1)} \le \frac{4\pi}{16T} < \frac{1}{T}$ from E with confidence 3/4.

To apply the ℓ instances of conditional applications of powers of V; $\{V^{2^k}\}_{k=0}^{\ell-1}$, we apply ℓ different 16α-approximations of $e^{iHt} \otimes \mathbb{1}_{2^c}$ (using the fast forwarding) where each such application works on the state plus its own ancilla register initialized to 0 (as in Definition 1). We get that $\beta \le 16\alpha\ell \le 16\alpha \log(32T)$). □

Corollary 1. $\mathsf{FF}_{\text{exp}} \subseteq \mathsf{SEEM}_{\text{exp}}$

Proof. For any $\beta = O\left(\frac{1}{\text{poly}(n)}\right)$, a Hamiltonian $H \in \mathsf{FF}_{\text{exp}}$ can be FF for some $T = O(2^{\text{poly}(n)})$, with $\alpha = \frac{\beta}{16n\log(32T)} = O\left(\frac{1}{\text{poly}(n)}\right)$. Hence, by Claim 2, H is $(1 - e^{-n/18}, 1/T, \beta)$-SEEM, and therefore it is $(\eta, 1/T, \beta)$-SEEM for any $\eta, \beta = O\left(\frac{1}{\text{poly}(n)}\right)$. We conclude that $H \in \mathsf{SEEM}_{\text{exp}}$. □

We now prove that SEEM implies FF with the desired parameters:

Claim 3. Let H be an n qubit Hamiltonian with $\|H\| \le 1$ which is $(\eta, \delta E, \beta)$-SEEM for $\eta > 1/2$. Let $T\delta E < \frac{\pi}{2}$, then H is also $(T,\ 2\eta \sin(\delta E T) + 2(1 - \eta + \beta))$-FF.

Proof. The idea of the proof is to apply the unitary $\widetilde{U}_{\text{SEEM}}$ approximating the measurement of the energy, which exists since the Hamiltonian can be super-efficiently measured, by Definition 2. Then, based on the output E' of this measurement, written on the quantum register, multiply the state by the phase $e^{-iE't}$ (denote this by the gate V), and finally apply the inverse of the approximated measurement unitary. Let $|\alpha\rangle = |\psi_E\rangle \otimes |0\rangle$. First we consider the exact measurement with no demolition ($\beta = 0$),

U_{SEEM}; notice that it commutes with H:

$$\begin{aligned}\left\|\left(U^\dagger_{\mathrm{SEEM}} V U_{\mathrm{SEEM}} - e^{-iHt}\otimes \mathbb{1}_{\mathcal{W}}\right)|\alpha\rangle\right\| &= \left\|\left(U^\dagger_{\mathrm{SEEM}} V U_{\mathrm{SEEM}} - U^\dagger_{\mathrm{SEEM}}\left(e^{-iHt}\otimes \mathbb{1}_{\mathcal{W}}\right)U_{\mathrm{SEEM}}\right)|\alpha\rangle\right\| \\ &= \left\|\left(V U_{\mathrm{SEEM}} - \left(e^{-iHt}\otimes \mathbb{1}_{\mathcal{W}}\right)U_{\mathrm{SEEM}}\right)|\alpha\rangle\right\|,\end{aligned} \tag{37}$$

where the Hilbert space of the work/output register is denoted by $\mathcal{W}$. On a specific eigenvector ψ_E:

$$\begin{aligned}&\left\|V U_{\mathrm{SEEM}}|\psi_E,0,0\rangle - \left(e^{-iHt}\otimes \mathbb{1}_{\mathcal{W}}\right)U_{\mathrm{SEEM}}|\psi_E,0\rangle\right\| = \left\|\sum_{E'} a_{E'}(e^{-iE't}-e^{-iEt})|\psi_E,E',g(E')\rangle\right\| \\ &= \left\|\sum_{E':|E'-E|\le\delta E} a_{E'}(e^{-iE't}-e^{-iEt})|\psi_E,E',g(E')\rangle + \sum_{E':|E'-E|>\delta E} a_{E'}(e^{-iE't}-e^{-iEt})|\psi_E,E',g(E')\rangle\right\| \\ &\le 2\eta\sin(\delta E t) + 2(1-\eta),\end{aligned} \tag{38}$$

where the last inequality is correct for $t \le \pi/2\delta E$. Notice that the above holds for any state $|\psi\rangle = \sum_{c_E} c_E|\psi_E\rangle$, using the fact that both U_{SEEM} and V leave the left register in tact. The proof follows since we have $\left\|\widetilde{U}^\dagger_{\mathrm{SEEM}} V \widetilde{U}_{\mathrm{SEEM}} - U^\dagger_{\mathrm{SEEM}} V U_{\mathrm{SEEM}}\right\| \le 2\beta$. □

Corollary 2. Let H be a normalized Hamiltonian on n qubits, which is $(\eta,\delta E,\beta)$-SEEM for $\eta > 1/2$ and $\beta < \pi/2$. Then H is also $(\beta/\delta E, 2n\beta + O(2^{-\mathrm{poly}(n)})$-FF.

Proof. Using Lemma 1 with $m = n-1$ we reach an $(1 - e^{-\frac{(n-1)\eta}{2}\left(1-\frac{1}{2\eta}\right)^2}, \delta E, (n-1)\beta)$-SEEM. Now choose $T = \beta/\delta E$ and since $T\delta E = \beta < \pi/2$ we can apply claim 3. The FF error α according to Claim 3 is bounded by $2n\beta + 2^{-\mathrm{poly}(n)}$.

□

Corollary 3. $\mathsf{SEEM}_{\mathrm{exp}} \subseteq \mathsf{FF}_{\mathrm{exp}}$

Proof. Let $H \in \mathsf{SEEM}_{\mathrm{exp}}$, $\delta E = \Omega(2^{-\mathrm{poly}(n)})$. We choose T to be any exponentially growing function such that $T\delta E$ decays faster than any polynomial (say, $T = \frac{1}{\delta E^{0.99}}$). Let $\alpha = O\left(\frac{1}{\mathrm{poly}(n)}\right)$ be a goal parameter for the fast forwarding. By assumption, H is $(\eta = 2/3, \delta E, \beta = \alpha/3n = O\left(\frac{1}{\mathrm{poly}(n)}\right)) -$SEEM. From corollary 2, H is also $(\beta/\delta E, \alpha)$-FF. By our choice of T, it is thus (T,α)-FF. Since this holds for any inverse polynomial α, we have $H \in \mathsf{SEEM}_{\mathrm{exp}}$. □

This completes the proof of theorem 2.

Supplementary Note 6: Proof of theorem 3 (FF Commuting H)

Theorem 3. If H is an n qubit normalized commuting $k-$local Hamiltonian, with $k = O(\log(n))$, then it can be (T,α)-fast forwarded with $T = 2^{O(n)}$ and arbitrary exponentially small α.

Proof. Since the terms H_j commute, we have $e^{-iHt} = \prod_j e^{-iH_jt}$. It thus suffices to be able to implement e^{-iH_jt} for t exponentially large, with the required exponentially small error. Let U_j be a unitary matrix which diagonalizes H_j, $D_j = U_jH_jU_j^\dagger$. Since U_j acts non-trivially on k qubits at most, all entries of U_j's eigenvalues and eigenvectors can be efficiently calculated classically to within exponential accuracy (e.g., [8,9]). Standard quantum computation techniques [3] can be used to apply e^{-iH_jt} to within any desired exponentially good accuracy. □

Supplementary Note 7: Proof of theorem 4 (FF Quadratic Fermion H)

Theorem 4. Let H be a quadratic Hamiltonian of n Fermions with poly(n) modes. H can be (T,α)-fast forwarded with $T = 2^{\Omega(n)}$ and arbitrary inverse polynomial α.

Quadratic Fermion Hamiltonians are defined as follows

$$H = \sum_{i,j}^{m} A_{i,j} a_i^\dagger a_j + \frac{1}{2}\sum_{ij} B_{i,j} a_i a_j + \frac{1}{2}\sum_{i,j} B_{j,i}^* a_i^\dagger a_j^\dagger \qquad A = A^\dagger, B = B^\dagger, \tag{39}$$

where $a_i, a_i^\dagger$ are the annihilation and creation operators respectively

$$\{a_i, a_j\} = 0 \quad \left\{a_i^\dagger, a_j^\dagger\right\} = 0 \quad \left\{a_i, a_j^\dagger\right\} = \delta_{i,j}. \tag{40}$$

The proof idea is to efficiently "diagonalize" the Hamiltonian by the Bogoliubov transformation [10,11] to the form $H = \sum_i \lambda_i b_i^\dagger b_i + \mathrm{tr}(A)/2$. The operators $b_i, b_i^\dagger$ are called quasiparticle annihilation and creation operators respectively, and they inherit the commutation/anti commutation relations of $a_i, a_i^\dagger$ as in Supplementary Equations (40). Additionally, the number operator $b_i^\dagger b_i$ has integer eigenvalues. Fast forwarding is enabled by efficiently evolving the system under $H' = \sum_i (\lambda_i t \mod 2\pi) b_i^\dagger b_i$ for one time unit, and by adding a global phase $t \cdot \mathrm{tr}(A)/2$.

Claim 4. Let $\mathbf{a}$ be a column vector whose j^{th} coordinate is a_j and let $\mathbf{a}^\dagger$ be a column vector whose j^{th} coordinate is $a_j^\dagger$. The Hamiltonian H can be written as

$$H = \frac{1}{2}\begin{pmatrix} \overline{\mathbf{a}^\dagger} & \overline{\mathbf{a}} \end{pmatrix}\begin{pmatrix} A & B^* \\ B & -A^* \end{pmatrix}\begin{pmatrix} \mathbf{a} \\ \mathbf{a}^\dagger \end{pmatrix} + \frac{1}{2}\mathrm{tr}(A) \tag{41}$$

Here, the overline indicates a matrix transposition, i.e., $\overline{\mathbf{a}^\dagger}, \overline{\mathbf{a}}$ are the row vectors corresponding to $\mathbf{a}^\dagger, \mathbf{a}$ respectively.

Proof. The proof relies on the hermiticity of A, B and the anticommutation relations. For $i \neq j$

$$\sum_{i\neq j} A_{i,j} a_i^\dagger a_j = \frac{1}{2}\sum_{i\neq j}\left(A_{i,j} a_i^\dagger a_j - A_{i,j} a_j a_i^\dagger\right) = \frac{1}{2}\left(\sum_{i\neq j} A_{i,j} a_i^\dagger a_j - \sum_{j\neq i} A_{j,i} a_i a_j^\dagger\right) = \frac{1}{2}\sum_{i\neq j}(A_{i,j} a_i^\dagger a_j - A_{i,j}^* a_i a_j^\dagger) \tag{42}$$

For $i = j$,

$$\sum_i A_{i,i} a_i^\dagger a_i = \frac{1}{2}\sum_i A_{i,i} a_i^\dagger a_i + \frac{1}{2}\sum_i A_{i,i}(1 - a_i a_i^\dagger) = \frac{1}{2}\left(\sum_i A_{i,i} a_i^\dagger a_i - \sum_i A_{i,i}^* a_i a_i^\dagger + \mathrm{tr}(A)\right) \tag{43}$$

Reorganizing H as a block matrix concludes the proof. □

Claim 5. The traceless part of the Hamiltonian can be diagonalized:

$$H = \frac{1}{2}\begin{pmatrix} \overline{\mathbf{a}^\dagger} & \overline{\mathbf{a}} \end{pmatrix} U D U^\dagger \begin{pmatrix} \mathbf{a} \\ \mathbf{a}^\dagger \end{pmatrix} + \frac{1}{2}\mathrm{tr}(A) \tag{44}$$

where D is a real diagonal matrix s.t. $D_{j,j} = -D_{j+n,j+n}$ and U is unitary. Furthermore, there exist matrices V_1, V_2 s.t. U is a block matrix in the form $U = \begin{pmatrix} V_1 & V_2^* \\ V_2 & V_1^* \end{pmatrix}$

Proof. Note the following symmetry of the traceless matrix $\mathcal{H}$:

$$\mathcal{H} \equiv \begin{pmatrix} A & B^\dagger \\ B & -A^* \end{pmatrix} \qquad \mathcal{H}^\dagger = \mathcal{H}, \qquad \tau\mathcal{H}\tau = -\mathcal{H}^*, \qquad \tau \equiv \begin{pmatrix} 0 & \mathbb{1}_m \\ \mathbb{1}_m & 0 \end{pmatrix} \tag{45}$$

The symmetry of $\mathcal{H}$ implies a symmetry on its eigenvectors, which are the column vectors of U.

$$\mathcal{H}\begin{pmatrix} v_1 \\ v_2 \end{pmatrix} = \lambda \begin{pmatrix} v_1 \\ v_2 \end{pmatrix} \Rightarrow \mathcal{H}\begin{pmatrix} v_2^* \\ v_1^* \end{pmatrix} = -\lambda \begin{pmatrix} v_2^* \\ v_1^* \end{pmatrix} \tag{46}$$

Hence if either $v_1 \neq v_2^*$ or $v_2 \neq v_1^*$, then the eigenvectors come in pairs: $w, \tau w^*$. In the case $v_1 = v_2^*$ and $v_2 = v_1^*$, we get that $\lambda = 0$. Since there is an even number of eigenvectors of the first case, and the dimension of the subspace is $2m$, there's also an even number of eigenvectors of the second case, with eigenvalue 0. Picking a pair of orthogonal eigenvectors w_1, w_2, with eigenvalue 0, we can span the subspace they define using $w_1' = w_1 + iw_2$ and $w_2' = w_1 - iw_2$, and we get that $w_1' = \tau w_2'^*$. Hence all eigenvectors of $\mathcal{H}$ come in pairs $w, \tau w^*$. By placing one eigenvector of the ith pair in column i of U and the other eigenvector in column $m+i$, U takes the desired form. Additionally, the column placement forces $D_{j,j} = -D_{j+m,j+m}$. □

Claim 6. By defining

$$\begin{pmatrix} \mathbf{b} \\ \mathbf{b}^\dagger \end{pmatrix} = U^\dagger \begin{pmatrix} \mathbf{a} \\ \mathbf{a}^\dagger \end{pmatrix}. \tag{47}$$

The Hamiltonian takes the form

$$H = 2\sum_{i=1}^{m} b_i^\dagger b_i D_{i,i} + \frac{1}{2}\operatorname{tr}(A) \tag{48}$$

The new operators obey the anti-commutation relations of Fermions. In addition, the eigenvalues of $b_i^\dagger b_i$ either 0 or 1.

Proof. From Supplementary Equations (47) and the definition of U,

$$b_i = \sum_j (V_1^\dagger)_{i,j} a_j + (V_2^\dagger)_{i,j} a_j^\dagger \tag{49}$$

$$b_i^\dagger = \sum_j (\overline{V}_2)_{i,j} a_j + (\overline{V}_1)_{i,j} a_j^\dagger \tag{50}$$

Hence $b_i^\dagger$ is indeed the Hermitian conjugate of b_i. Additionally, one can see that $\begin{pmatrix} \overline{\mathbf{b}^\dagger} & \overline{\mathbf{b}} \end{pmatrix} = \begin{pmatrix} \overline{\mathbf{a}^\dagger} & \overline{\mathbf{a}} \end{pmatrix} U$. Next we show that the transformation is canonical, i.e., the anticommutation relations are preserved:

$$\begin{aligned} \{b_i, b_j\} &= \left\{ \sum_{k=1}^m U_{k,i}^* a_k + \sum_{k=1}^m U_{k+m,i}^* a_k^\dagger, \sum_{\ell=1}^m U_{\ell,j}^* a_\ell + \sum_{\ell=1}^m U_{\ell+m,j}^* a_\ell^\dagger \right\} \\ &= \sum_{k=1}^m \left(U_{k,i}^* U_{k+m,j}^* + U_{k+m,i}^* U_{k,j}^* \right) = \sum_{k=1}^m \left(U_{k,i}^* U_{k,j+m} + U_{k+m,i}^* U_{k+m,j+m} \right) = 0 \end{aligned} \tag{51}$$

(we used the structure of U for the transition of the second line)

$$\left\{ b_i, b_j^\dagger \right\} = \left\{ \sum_{k=1}^m U_{k,i}^* a_k + \sum_{k=1}^m U_{k+m,i}^* a_k^\dagger, \sum_{\ell=1}^m U_{\ell,j} a_\ell^\dagger + \sum_{\ell=1}^m U_{\ell+m,j} a_\ell \right\} = \sum_{k=1}^m \left(U_{k,i}^* U_{k,j} + U_{k+m,i}^* U_{k+m,j} \right) = \delta_{i,j} \tag{52}$$

Finally, the eigenvalues of $b_j^\dagger b_j$ are 0 and 1, because $(b_j^\dagger b_j)^2 = b_j^\dagger b_j b_j^\dagger b_j = (1 - b_j b_j^\dagger) b_j^\dagger b_j = b_j^\dagger b_j$, similarly to the number operator $a_j^\dagger a_j$. □

We can now use the above claims to achieve FF. In Supplementary Equation (48), the modes b_i are independent, and in particular, the Hamiltonian is a sum of m commuting terms. Therefore, an evolution under H for time t can be implemented by

$$e^{iHt} = e^{-it\operatorname{tr}(A)/2} \prod_j e^{2iD_{jj} t b_j^\dagger b_j} \tag{53}$$

Since the eigenvalues of each term are $D_{j,j}t$ times an integer (using Claim 6) we have that if we replace $\mathcal{H}$ by a matrix with the same eigenvectors but with eigenvalues ($D_{j,j}t \mod 2\pi$), it will have the same evolution on any state, in other words if we define

$$H' = \frac{1}{2}\begin{pmatrix} \overline{\mathbf{a}^\dagger} & \overline{\mathbf{a}} \end{pmatrix} UD'U^\dagger \begin{pmatrix} \mathbf{a} \\ \mathbf{a}^\dagger \end{pmatrix} + \frac{1}{2}\operatorname{tr}(A) \tag{54}$$

$$D'_{i,j} = \delta_{i,j} D_{i,i} t \mod 2\pi \tag{55}$$

we get

$$e^{-iH'} = e^{-iHt} \tag{56}$$

Hence it is sufficient to simulate the evolution under H' to time $t = 1$. To do this we observe that Supplementary Equation (54) means that H' is a quadratic Hamiltonian in $\{a_i\}$ and $\{a_i^\dagger\}$, whose coefficients can be calculated by a classical computer in time polynomial in m to within exponential accuracy [8,9]. Assuming that we can implement any quadratic Hamiltonian of polynomial number of coefficients exactly, we need only apply H' for one time unit to fast forward H for exponential duration t, with arbitrary exponentially small α. However, the assumption that a quadratic Hamiltonian with general coefficients can be implemented exactly is not realistic; Assuming inverse polynomial error in each of the coefficients in the quadratic Hamiltonian results in an overall inverse polynomial error and thus would still lead to a fast forwarding procedure for exponential duration of time, but with inverse polynomial error α.

Supplementary Note 8: Proof of No generic FF

We now prove theorem 5.

Theorem 5. A generic procedure for ($T = 2^{(n^{1/c})}, \alpha = n^{-4/c}$)-fast forwarding a 2-sparse row-computable Hamiltonian, with $c > 1$, does not exist (unless BQP = PSPACE).

2-sparse row-computable Hamiltonians are those with at most two non-zero entries per row in the matrix representation of the Hamiltonian. These entries also need to be computed efficiently given the row index. Such Hamiltonians are known to be efficiently simulable by quantum circuits [12, 13].

BQP is the class of problems that can be solved in polynomial time by a quantum computer; PSPACE is the class of problems that can be solved in polynomial space by a classical (or quantum, this doesn't matter) computer. It is known that PSPACE contains BQP but it is strongly believed to be a strictly larger class.

The proof of the theorem is by using the procedure to solve the PSPACE-complete problem `OTHER END OF THIS LINE (OEOTL)` [14] in polynomial time. We recall the definition of the problem

Definition 3 (`OEOTL`). Let $G = (V, E)$ be a directed graph whose 2^n vertices are indexed by n bits vectors. G is given by two polynomial size classical circuits S and P, s.t. there is an edge from u to v only if $S(u) = v$ and $P(v) = u$ (Hence G contains only paths, cycles, or isolated vertices). Given such a G and given that the vertex 0^n has no incoming edge but has an outgoing edge, find the other end of the line that starts with 0^n.

The algorithm is as follows:

1. Let $v_0 = 0$, $H_0 = H$
2. For i=1 to $100n$
   (a) Check that 10 steps forward from v_{i-1} the end of the line is not reached; if it is, output it and exit.
   (b) Perform a $(1 - e^{-n/18}, 5^{-n}, 40n^{-2})$-SEEM on the state $|v_{i-1}\rangle$ under H_{i-1}.
   (c) Measure in the computational basis, denote the result by v_i. Let H_i be the original Hamiltonian H with the edge $(v_i - 1, v_i)$ removed.
3. Move 10 steps forward from the vertex reached. If the end of the line is found return it and exit. Otherwise, the algorithm fails.

Proof of correctness:
The idea of the proof is to make progress on the path, as follows: Starting from the first node v_0, we use the SEEM (stage 2b) to measure the energy with respect to H_0 (assume $\beta = 0$ for now). Due to the high accuracy of the measurement, the resulting state, conditioned on the measurement outcome, is close to an eigenstate. All eigenstates are symmetric around the middle of the path, hence the measurement in the computational basis in stage 2c, yields with good probability a vertex v_1 that is closer to the end of the path than to v_0 namely, the remaining path length is likely to be halved. We call this event a successful iteration, and show its probability is at least 1/10 if the path length is more than 10. An unsuccessful iteration does not increase the length of the path, it just doesn't succeed in shrinking by half. The vertex v_1 is now the next starting point, and H_1 is fixed to prevent going backwards by correcting H to not include the edge connecting v_1 to the previous vertex on the line. After n successful iterations, the vertex reached should be the end of the line. By Chernoff, the probability of at least n successful iterations with 1/10 success probability, out of $100n$ iterations is $\geq 1 - e^{-81n/20}$, which is exponentially close to one. If at some stage the length of the path is smaller than 10, the success probability may be smaller than 1/10, but the end of the line is found in stage 3. An analysis of $\beta > 0$ concludes the proof.

Let $U_{\text{meas.}}$ be the measurement with $\beta = 0$, and confidence $\eta = 1 - e^{-n/18}$ with respect to H_i. Suppose the vertex found in the previous round is $v = v_i$, and consider applying $U_{\text{meas.}}$ to $|v, 0, 0\rangle$ where the additional two registers are the output and work registers. Denote the result of measuring the two additional registers by ε_j, g. Let f be the function s.t. $\psi_{f(j)}$ is the eigenstate of H_i with energy closest to ε_j (the lower energy eigenstate if there is a tie). f is well defined since all eigenvalues of H_i within the relevant subspace have multiplicity 1. We omit adding an i index to f, and to the eigenstates/eigenvalues of H_i since both H and H_i in the relevant subspaces are Hamiltonians of paths, only the length of the path and the starting vertex change.

Claim 7. Let a_j be the amplitude of $\psi_{f(j)}$ after measuring ε_j, g. The expectation of $|a_j|^2$ over j, g satisfies: $\mathbb{E}_{j,g}\left(|a_j|^2\right) = \sum_{g,j} |a_j|^2 \Pr(\varepsilon_j, g) \geq \eta$.

Proof. Let f^{-1} be the preimage of f,

$$\begin{aligned}\mathbb{E}_{j,g}(|a_j|^2) &= \sum_{g,j} |a_j|^2 \Pr(\varepsilon_j, g) = \sum_{g,j} \Pr(\psi_{f(j)}|\varepsilon_j, g) \Pr(\varepsilon_j, g) = \sum_{g,j} \Pr(\varepsilon_j, g, \psi_{f(j)}) \\ &= \sum_k \Pr(\psi_k) \sum_{g,j:j\in f^{-1}(k)} \Pr(\varepsilon_j, g|\psi_k) \geq \eta\end{aligned} \tag{57}$$

The last inequality, is due to the η-confidence of the measurement, and that all measurement outcomes in the window δE around E_k are in $f^{-1}(k)$. □

Claim 8. Let $L_i = L - v_i \geq 10$ and $\ell_i = \left\lceil \frac{L_i}{2} \right\rceil$. The probability for a successful iteration, i.e., $v_{i+1} \geq v_i + \ell_i$ is at least 1/10 for the value of η.

Proof. After measuring ε_j, g, the state of the system is $a_j \left|\psi_{f(j)}\right\rangle + \sqrt{1 - |a_j|^2} \left|\psi^{\perp}_{f(j)}\right\rangle$. We define the Π_i to be a projection on the vertices $v \geq v_i + \ell_i$. The symmetry of the eigenstates around the middle of the path implies that $2\left\|\Pi_i \left|\psi_{f(j)}\right\rangle\right\|^2 + \frac{2}{L_i+1} \geq 1$, therefore $\frac{1}{2} - \frac{1}{L_i+1} \leq \left\|\Pi_i \left|\psi_{f(j)}\right\rangle\right\|^2 \leq \frac{1}{2}$.

$$\begin{aligned}\Pr(v_{i+1} \geq v_i + \ell_i|\varepsilon_j, g) &= \left\|\Pi_i \left(a_j \left|\psi_{f(j)}\right\rangle + \sqrt{1 - |a_j|^2} \left|\psi^{\perp}_{f(j)}\right\rangle\right)\right\|^2 \\ &\geq |a_j|^2 \left\|\Pi_i \left|\psi_{f(j)}\right\rangle\right\|^2 + \left(1 - |a_j|^2\right) \left\|\Pi_i \left|\psi^{\perp}_{f(j)}\right\rangle\right\|^2 - 2|a_j| \sqrt{1 - |a_j|^2} \left\|\Pi_i \left|\psi_{f(j)}\right\rangle\right\| \left\|\Pi_i \left|\psi^{\perp}_{f(j)}\right\rangle\right\| \\ &\geq |a_j|^2 \left(\frac{1}{2} - \frac{1}{L_i+1}\right) - |a_j| \sqrt{2 - 2|a_j|^2}\end{aligned} \tag{58}$$

Using the inequality $x\sqrt{2 - 2x^2} \leq 99(1 - x^2) + 0.01$ for $0 \leq x \leq 1$, we bound the probability by

$|a_j|^2 \left(99.5 - \frac{1}{L_i+1}\right) - 99.01$. Finally, we average over all g, j:

$$\begin{aligned} \Pr\left(v_{i+1} \geq v_i + \ell_i\right) &\geq \sum_{g,\varepsilon_j} \Pr\left(v_{i+1} \geq v_i + \ell_i | \varepsilon_j, g\right) \Pr(\varepsilon_j, g) \geq \mathbb{E}_{j,g}\left(|a_j|^2\right)\left(99.5 - \frac{1}{L_i+1}\right) - 99.01 \\ &\geq \eta\left(99.5 - \frac{1}{L_i+1}\right) - 99.01 \end{aligned} \tag{59}$$

With $\eta = 1 - e^{-n/18}$, the probability for a successful iteration is at least $1/10$ for $L_i > 10$. □

Claim 9. When the algorithm applies the SEEM with $\beta = 0$, it succeeds with probability at least $1 - e^{-81n/20}$.

Proof. Consider the $100n$ iterations in the protocol, when if the protocol had ended before completing all iterations, the iteration is simply idle. An iteration i is declared "successful" if either it is idle, or if not, the length of the path had been halved during step 2(c) of this iteration. By this definition and by Claim 8, the probability of the i^{th}iteration to be successful is $> 1/10$, even when we condition on what happened in previous iterations. Let X be the number of successful iterations out of the $100n$ iterations. We want to bound the probability that $X > n$ from below. We note that this probability is bounded from below by the corresponding probability for the number Y of successful iterations when we have $100n$ i.i.d Bernoulli variables, each with probability exactly $1/10$ for success. For i.i.d variables we can use the Chernoff bound,

$$\Pr(Y \leq (1-\delta)\mu) \leq e^{-\delta^2\mu/2}. \tag{60}$$

where μ is the expectation of Y, and we have here $\mu = 10n$. Setting $(1-\delta)\mu = n$, $\delta = 9/10$, we have $\Pr(Y > n) \geq 1 - e^{-81n/20}$. This means that $Pr(X > n)$, the probability for at least n successful iterations, is at least $1 - e^{-81n/20}$. After n successful iterations the path length must have reached below 10 since $\frac{L}{2^n} \leq 1$, and the algorithm succeeds in finding the end of the line. □

We analyze what is the success probability of the algorithm with $\beta > 0$. To this end, consider first a unitary version of the above algorithm, still with $\beta = 0$, where the only measurement is at the end. In stage 2c, we copy (using cNOTs) the result v_i to a separate register in every iteration instead of measuring. In stage 2b we apply the super efficient energy measurement, where the Hamiltonian is conditioned on the copy of v_i. The outcome and the garbage are written on a separate register in every iteration. At the end of the algorithm, an indicator qubit is set to 1 if the algorithm found the end of the line and 0 otherwise. In this version the algorithm is unitary, and the only difference between $\beta = 0$ and $\beta > 0$ cases are the $100n$ instances of SEEMs. Thus, at the end of the algorithm, just before measuring, the state with $\beta > 0$ SEEMs (denoted $\widetilde{\xi}$) deviates at most by $100\beta n$ from the state in which $\beta = 0$ SEEMs were used (denoted ξ). Let Π be the projection on a successful outcome of the algorithm, i.e., the indicator qubit is 1. We bound the algorithm success probability for $\beta > 0$:

$$\left\|\Pi\left|\widetilde{\xi}\right\rangle\right\|^2 = \left\|\Pi\left|\xi\right\rangle + \Pi\left(\left|\widetilde{\xi}\right\rangle - \left|\widetilde{\xi}\right\rangle\right)\right\|^2 \geq (\|\Pi\left|\xi\right\rangle\| - 100\beta n)^2 \geq \|\Pi\left|\xi\right\rangle\|^2 - 200\beta n \tag{61}$$

Hence the success probability is reduced by $200\beta n$. Since $\beta = 40n^{-2}$ the success probability is polynomially close to 1.

The main contribution to the time complexity of the algorithm is from the $100n$ rounds of step 3. The SEEM in each round calls $O(\log n)$ times to the fast forwarding procedure. Hence the time complexity is polynomial in n.

We conclude the proof of Theorem 5 by relaxing the demand for the generic FF procedure. Consider a generic fast forwarding procedure for n qubit Hamiltonians with parameters $T = 2^{(n^{1/c})}$, and $\alpha = n^{-4/c}$ with $c > 1$. This procedure is weaker than a generic fast forwarding procedure with parameters $T = 5^n$ and $\alpha = n^{-4}$ used in the proof, however one can use the weaker procedure to $(5^n, n^{-4})$-FF any Hamiltonian.

Given an n qubit Hamiltonian H, one can define an $m = (3n)^c$ qubit Hamiltonian $H' = H \otimes \mathbb{1}_{2^{m-n}}$. H' is still 2-sparse row-computable, therefore it can be FF using the weaker procedure for $T = 2^{(m^{1/c})} \geq 5^n$ and with $\alpha = m^{-4/c} = (3n)^{-4} < n^{-4}$ (polynomial complexity in m is also polynomial in n). Hence `OEOTL` can be solved efficiently using the weaker generic FF procedure. □

Supplementary Note 9: Uncertainty relations and metrology

The results presented here are related in various ways to the problem of highly sensitive measurements in metrology. A typical problem in sensing or quantum metrology (see e.g. [4, 15]), is to estimate the phase φ of an unknown phase shifter (i.e., a black box). The canonical setting is a Mach-Zehnder interferometer. Assuming the optical paths are equal, the Fock state of the probe before the measurement is $\cos(\varphi/2)\left|1\right\rangle_a \left|0\right\rangle_b + i\sin(\varphi/2)\left|0\right\rangle_a \left|1\right\rangle_b$, where a, b denote the two spatial modes. φ is estimated by measuring which of the paths the probe had taken (and taking statistics over many experiments). Classically, one can improve the accuracy (standard deviation here) by a factor of $\sqrt{n}$ (achieving the standard quantum limit) by repeating the experiment n times independently. A more efficient parameter estimation than this straightforward approach is categorized as super-sensing. Using n entangled photons in the "NOON state" [16–18] $\left|n\right\rangle_a \left|0\right\rangle_b + \left|0\right\rangle_a \left|n\right\rangle_n$ the distinguishability improves by a factor of n. When the Hamiltonian is accessed as a black box, this $1/n$ scaling is optimal. This bound is referred to as the Heisenberg limit [4, 15, 19, 20].

An interesting observation is that in this example, the experiment time is a constant, i.e., independent of the number of photons. This is because in physical reality, there is no limit on the number of photons that can reside in a Hamiltonian confined to a volume in space (e.g., a phase shifter). We note that this is not allowed in both the computational models of [1] and in our definition of unitary energy measurement - the Hamiltonian in these models is applied to one system at a time (we can call this "the single probe assumption"). When simulating the above NOON experiment in our computational model, we will get a factor of n in the computational complexity, and thus the complexity of the measurement will become $n\tau$ where τ is the time a probe spends inside the phase shifter. In this setting of estimating the phase of the phase shifter, the Heisenberg limit coincides with the TEUR, since the TEUR then reads

$$n\tau \cdot \Delta\varphi/\tau = n \cdot \Delta\varphi \geq 1. \tag{62}$$

In the general case, one is interested in parameter estimation of a Hamiltonian which is a known operator function depending on some set of unknown parameters. In these cases, some partial knowledge about the eigenvalues is given; The question of whether it is possible to obtain violations of the cTEUR when the Hamiltonian is not accessed through a black-box remains to be investigated.

References

[1] Aharonov, Y., Massar, S. & Popescu, S. Measuring energy, estimating hamiltonians, and the time-energy uncertainty relation. Phys. Rev. A **66**, 052107 (2002).

[2] Aharonov, Y. & Bohm, D. Time in the quantum theory and the uncertainty relation for time and energy. Physical Review **122**, 1649–1658 (1961).

[3] Nielsen, M. A. & Chuang, I. L. Quantum Computation and Quantum Information (Cambridge University Press, 2000).

[4] Giovannetti, V., Lloyd, S. & Maccone, L. Quantum metrology. Physical review letters **96**, 010401 (2006).

[5] Rotman, J. An introduction to the theory of groups, vol. 148 (Springer Science & Business Media, 2012).

[6] Shor, P. W. Polynomial-time algorithms for prime factorization and discrete logarithms on a quantum computer. SIAM J. Comp. **26**, 1484–1509 (1997).

[7] Kitaev, A. Y., Shen, A. & Vyalyi, M. N. Classical and quantum computation. 47 (American Mathematical Soc., 2002).

[8] Pan, V. Y. & Chen, Z. Q. The complexity of the matrix eigenproblem. In Proceedings of the thirty-first annual ACM symposium on Theory of computing, 507–516 (ACM, 1999).

[9] Armentano, D., Beltrán, C., Bürgisser, P., Cucker, F. & Shub, M. A stable, polynomial-time algorithm for the eigenpair problem. Preprint at http://arxiv.org/abs/1505.03290 (2015).

[10] Blaizot, J.-P. & Ripka, G. Quantum theory of finite systems, vol. 3 (MIT Press Cambridge, 1986).

[11] Shchesnovich, V. The second quantization method for indistinguishable particles (lecture notes in physics). Preprint at http://arxiv.org/abs/1308.3275 (2013).

[12] Aharonov, D. & Ta-Shma, A. Adiabatic quantum state generation and statistical zero knowledge. In Proceedings of the thirty-fifth annual ACM symposium on Theory of computing, 20–29 (ACM, 2003).

[13] Berry, D. W., Childs, A. M., Cleve, R., Kothari, R. & Somma, R. D. Exponential improvement in precision for simulating sparse hamiltonians. In Proceedings of the 46th Annual ACM Symposium on Theory of Computing, 283–292 (ACM, 2014).

[14] Papadimitriou, C. H. On the complexity of the parity argument and other inefficient proofs of existence. Journal of Computer and system Sciences **48**, 498–532 (1994).

[15] Giovannetti, V., Lloyd, S. & Maccone, L. Advances in quantum metrology. Nature Photonics **5**, 222–229 (2011).

[16] Yurke, B. Input states for enhancement of fermion interferometer sensitivity. Physical review letters **56**, 1515 (1986).

[17] Sanders, B. C. Quantum dynamics of the nonlinear rotator and the effects of continual spin measurement. Physical Review A **40**, 2417 (1989).

[18] Lee, H., Kok, P. & Dowling, J. P. A quantum rosetta stone for interferometry. Journal of Modern Optics **49**, 2325–2338 (2002).

[19] Zwierz, M., Pérez-Delgado, C. A. & Kok, P. General optimality of the heisenberg limit for quantum metrology. Physical review letters **105**, 180402 (2010).

[20] Zwierz, M., Pérez-Delgado, C. A. & Kok, P. Ultimate limits to quantum metrology and the meaning of the heisenberg limit. Physical Review A **85**, 042112 (2012).
